# High-Resolution Mass Spectrometry Driven Discovery of Peptidic Danger Signals in Insect Immunity

**DOI:** 10.1371/journal.pone.0080406

**Published:** 2013-11-26

**Authors:** Arton Berisha, Krishnendu Mukherjee, Andreas Vilcinskas, Bernhard Spengler, Andreas Römpp

**Affiliations:** 1 Institute of Inorganic and Analytical Chemistry, Justus Liebig University, Giessen, Germany; 2 Institute of Phytopathology and Applied Zoology, Justus Liebig University, Giessen, Germany; Louisiana State University, United States of America

## Abstract

The ‘danger model’ is an alternative concept for immune response postulating that the immune system reacts to entities that do damage (danger associated molecular patterns, DAMP) and not only to entities that are foreign (pathogen-associated molecular patterns, PAMP) as proposed by classical immunology concepts. In this study we used *Galleria mellonella* to validate the danger model in insects. Hemolymph of *G. mellonella* was digested with thermolysin (as a representative for virulence-associated metalloproteinases produced by humanpathogens) followed by chromatographic fractionation. Immune-stimulatory activity was tested by measuring lysozyme activity with the lytic zone assays against *Micrococcus luteus* cell wall components. Peptides were analyzed by nano-scale liquid chromatography coupled to high-resolution Fourier transform mass spectrometers. Addressing the lack of a genome sequence we complemented the rudimentary NCBI protein database with a recently established transcriptome and *de novo* sequencing methods for peptide identification. This approach led to identification of 127 peptides, 9 of which were identified in bioactive fractions. Detailed MS/MS experiments in comparison with synthetic analogues confirmed the amino acid sequence of all 9 peptides. To test the potential of these putative danger signals to induce immune responses we injected the synthetic analogues into *G. mellonella* and monitored the anti-bacterial activity against living *Micrococcus luteus*. Six out of 9 peptides identified in the bioactive fractions exhibited immune-stimulatory activity when injected. Hence, we provide evidence that small peptides resulting from thermolysin-mediated digestion of hemolymph proteins function as endogenous danger signals which can set the immune system into alarm. Consequently, our study indicates that the danger model also plays a role in insect immunity.

## Introduction

A prerequisite for the evolution of Metazoa was the ability to discriminate between self and nonself. This power of distinction is mediated by a functioning immune system which encompasses all endogenous mechanisms and molecules contributing to host defense against microbes and parasites.

Vertebrate and invertebrate animals share the ancient innate immunity mediating discrimination between infectious nonself and noninfectious self by a limited number of germ line encoded receptors which bind to molecules common among pathogens, but absent from the host such as microbial cell wall components, particularly, bacterial lipopolysaccharides (LPS), peptidoglycans, and fungal ß-1,3-glucans [1]. This binding of receptor proteins to pathogen-associated molecular patterns (PAMPs) differs from the highly specific recognition of a wide range of antigens by a large and diverse spectrum of somatically rearranged receptors of T and B cells which represent an evolutionary novelty of vertebrates. Their adaptive or acquired immunity relies on antibody-based immunological memory of infection [2]. Janewaýs model claiming that activation of the immune system requires recognition of PAMPs has become a paradigm, particularly in insect immunity [3]. Binding of PAMPs to corresponding pattern recognition receptors results in activation of immune responses including both cellular mechanisms such as phagocytosis or multicellular encapsulation of microbes, and humoral defences such as the rapid synthesis of antimicrobial peptides and proteins [4].

Matzinger has challenged immunological research by introducing her danger model, which is based on the idea that the immune system is more concerned with entities that do damage than with those that are foreign [5]. Her alternative model explains recognition of foreign threat by molecules released from damaged cells or wounded tissues whose binding to corresponding pattern recognition receptors results in activation of immune responses. Such damage-associated molecular patterns (DAMPs) function as danger signals which can set the immune system into alarm [6]. First evidence for the presence of immunity-related danger signals has been reported from insects, particularly in the larvae of the greater wax moth *Galleria mellonella* [7]. The latter have in between emerged as powerful model hosts for pathogens infecting insects or humans [8]. This study elucidated that activation of innate immune responses in *G. mellonella* does not necessarily require PAMPs, the presence of microbial enzymes, particularly metalloproteinases of the M4 family with thermolysin as the prototype, in the hemolymph is sufficient to produce DAMPs [7]. Members of the thermolysin-family encompass prominent virulence factors and toxins of human pathogens such as aureolysin, bacillolysin and pseudolysin which have been implicated to be responsible for increase of vascular permeability, hemorrhagic edema, sepsis and necrotic tissue destruction [9]. Thermolysin-mediated digestion of hemolymph proteins in *G. mellonella* results in formation of small peptidic fragments smaller than 3 kDa which elicit innate immune responses that are qualitatively (spectrum of immune-related proteins secreted within the hemolymph) and quantitatively (expression levels of antimicrobial peptides) comparable with the response to injected bacterial lipopolysaccharide (LPS), a widely used microbial elicitor of innate immune responses [10].

Mass spectrometry (MS) is a powerful and universal tool for the analysis and identification of proteins and peptides. Complex biological samples are usually separated by liquid chromatography or gel electrophoresis prior to MS analysis. The MS-based identification of peptides and proteins from *G. mellonella* is hindered by fact that the genome is not sequenced and thus protein databases are only rudimentary. Traditional database search approaches using SEQUEST [11] or Mascot [12] do not result in satisfactory results. To compensate for this impediment we have recently subjected the immunity-related transcriptome of *G. mellonella* to next generation sequencing using the Roche 454-FLX platform combined with traditional Sanger sequencing to obtain a comprehensive immune gene repertoire [13]. In addition, we have developed a method for *de novo* sequencing of peptides isolated from *G. mellonella* hemolymph [14]. The reliability of peptide identification can be significantly improved by using high resolution and accurate measurements [15], [16]. The highest mass resolution is achieved by Fourier Transform mass spectrometers based on orbital trapping [17] or ion cyclotron resonance [18], [19]. A method that simplifies *de novo* sequencing is the composition based sequencing (CBS) approach which takes advantage of accurate mass measurements [20]. An example for CBS analysis of the tree frog H. savigni with unknown genome sequence was recently reported [21].

The application of high-resolution mass spectrometry enabled the identification of a number of hemolymph protein fragments resulting from thermolysin-mediated hydrolysis of hemolymph. Their putative function as danger signals was verified using synthetic analogues which were injected into *G. mellonella* larvae in order to test their capacity to elicit humoral immune responses. As in our previous studies [7], [10] we used freeze-dried or living *Micrococcus luteus* bacteria as indicator organisms in lytic zone or inhibition zone assays, respectively.

## Materials and Methods

### Insect rearing and sample preparation

*G. mellonella* larvae were reared on an artificial diet (22% maize meal, 22% wheat germ, 11% dry yeast, 17.5% bee wax, 11% honey and 11% glycerin) at 32°C in darkness. For collecting the hemolymph the larvae were first cooled at 4°C for 15 min and the prolegs were pierced with a sterile needle. Hemolymph melanisation was prevented by the use of few crystals of phenylthiourea. Cell-free hemolymph was degraded with thermolysin (*Sigma*, Taufkirchen, Germany) with an end concentration of 1 mg/mL in sterile bidistilled water. The mixture was incubated for 1h at 36°C on a rotary shaker. These parameters (moderate enzyme concentration and short incubation time) were chosen in order to mimic the *in vivo* conditions. Thermolysin-degraded fragments smaller than 3 kDa were obtained gradually by centrifugation at 7000 x g for 4-6h at 4°C using Centricon centrifugal concentrators Centricon-30, Centricon-10 and Centricon-3 (*Millipore*, Billerica, Massachusetts, USA), respectively.

### Fractionation of hemolymph sample

Separation of the thermolysin-digested hemolymph sample (< 3 kDa) was realized by an *Agilent 1200 RP* HPLC system (*Agilent*, Waldbronn, Germany) with a *Symmetry* C18 4.6×250 mm column at a flow-rate of 1 mL/min. The injection volume of the sample was 100 µL. Separation was carried out using a 40 min gradient starting at 5% acetonitrile in water (v/v). After 15 min the solvent concentration of acetonitrile was raised up to 50% (v/v). After 40 min the concentration of acetonitrile was increased to 95% (v/v). The resulting fractions were collected manually in 30sec intervals, lyophilized and dissolved in 50 µL H_2_O. The fractions were injected in *Galleria* larvae and immune stimulation was determined by lytic zone assay, subsequently.

### Mass spectrometry

**NanoHPLC-ESI FTMS measurements.** The measurements of the samples were performed on a binary nanoHPLC system consisting of the units Switchos, Famos and Ultimate (*LCPackings/Dionex*, Idstein, Germany). Solvent A was water (HPLC grade, *Fluka,* Neu-Ulm, Germany) containing 2% acetonitrile (v/v) (Uvasol® grade, *Merck KGaA* Darmstadt, Germany) and 0.1% formic acid (v/v) (puriss p.a. for ms, *Fluka* Neu-Ulm, Germany). Solvent B was acetonitrile containing 20% water (v/v) and 0.08% formic acid (v/v). Separation of the hemolymph sample was carried out using a 77 min gradient. First B was increased from 0% to 10% in 10 min, subsequently increased to 30% in 30 min and then increased to 100% in 5 min and maintained for 11 min. The injection volumes were 0.3 µL for the hemolymph bulk sample and 1.2 to 5 µL for hemolymph fractions, respectively. After pre-concentration on a C18 PepMap trap column (5 mm×300 µm i.d.) the samples were separated on a fused silica C18 PepMap100 capillary column (150 mm×75 µm i.d.) (*Dionex*, Idstein, Germany) at 200 nL/min. The separation was monitored by a UV detector at 214 nm.

The nanoHPLC system was coupled to the mass spectrometer by a nanospray source. Pico-Tip® Emitter (*New Objective,* Woburn, MS, *USA*) were used as nanospray needles. The separated peptides were measured on a tandem mass spectrometer *(LTQ FT Ultra, Thermo Fisher Scientific GmbH*, Bremen, Germany) consisting of a linear ion trap (IT) and a Fourier Transform Ion Cyclotron Resonance (FTICR) mass spectrometer. For database searches high-resolution (R = 100.000 at m/z 400) survey spectra were measured on the FTICR with high mass accuracy (< 2 ppm). Fragment ion spectra were measured both with high resolution and in the ion trap. For analyses with manual *de novo* and the composition-based sequencing method precursor and fragment ions were solely measured with high resolution and high mass accuracy. *Collision induced dissociation* (CID) was used for fragmentation. MS/MS data was obtained on the FT also using the wide scan range (WSR) method with three microscans in order to compensate the loss of ions due to the time-of-flight effect of the FTICR mass spectrometer.

In addition, coupling of nanoHPLC with a linear ion trap / Fourier transform orbital trapping (IT-FTOT MS) mass spectrometer (*LTQ Orbitrap Discovery, Thermo Scientific GmbH*, Bremen, Germany) equipped with a nanospray ion source, was used. This mass spectrometer allowed, in addition to CID-fragmentation, the detection of low mass fragments by *higher-energy collisional dissociation* (HCD), providing additional information for peptide sequencing. Full scan and MS/MS spectra on the FTOT instrument were acquired with high resolution (R = 30.000 at m/z 400) and high mass accuracy (< 4 ppm). External calibration of both FT instruments was performed immediately before measurements according to the instructions of the manufacturer. All MS measurements were performed using the positive ion mode.

**Offline ESI FTMS measurements.** Offline nano ESI MS measurements of synthetic peptides were performed for validation of identified peptides using the orbitral trapping mass spectrometer. Pico tip emitters (5 µm i.d., *DNU-MS*, Berlin, Germany) were used for this task. The synthesized peptides were dissolved in 200 µL water/acetonitrile 50:50 (v/v) with 0.1% formic acid and diluted 1∶10 before measurement.

**MALDI FTMS measurements.** For matrix-assisted laser desorption/ionization (MALDI) measurements the commercial MALDI source of the orbital trapping mass spectrometer, equipped with a N_2_-Laser (337 nm), was used. Alpha-cyano-4-hydroxycinnamic acid (10 mg/mL) dissolved in acetonitrile/water 70:30 (v/v) with 0.1% trifluoroacetic acid was used as matrix. Two µL matrix solution were mixed with 1 µL sample on a stainless steel sample plate. The laser energy was set to 20 µJ and the crystal positioning system (CPS) was activated. MS and MS/MS experiments were performed with collision induced dissociation (CID) and higher-energy collisional dissociation (HCD) to obtain low-mass fragment ions.

### Data analysis / Identification of peptides

**Database search with standard database.** Data obtained from HPLC-MS measurements were analyzed with SEQUEST database search using the BioWorks V3.3.1 software (*Thermo Fisher Scientific*, Bremen, Germany). For bulk samples 5 HPLC-MS measurements were carried out and at least 2 measurements for each bioactive fraction. Protein sequence database entries for *G. mellonella* are very limited. Therefore all available database entries for *Galleria* were downloaded from the server of the “*National Center of Biotechnology Information*” NCBI (http://www.ncbi.nlm.nih.gov/). This database contained 162 protein sequence database entries. The database searches of all HPLC-MS measurements were carried out with following settings: precursor ion tolerance 5 ppm; fragment ion tolerance 1 Da. The following variable modifications were selected: phosphorylation on serine, threonine, and tyrosine, hydroxylation on proline, threonine and methionine and carbamidomethylation on cysteine. These modifications are considered in the software with a mass increment of the respective amino acid, for hydroxylation Δm = +15.99492, for phosphorylation Δm = +79.96633 and for carbamidomethylation Δm = +57.02146, respectively. Thermolysin cleaves at the N-terminal side of amino acids with hydrophobic or bulky side chains [22]. The amino acids alanine, isoleucine, leucine, phenylalanine, valine and methionine were selected as cleavage sites. The number of allowed missed cleavage sites was set to 10. Peptides with a *Peptide Probability* below 0.001 were considered as identified. False discovery rates (FDR) were calculated according to Nesvizhskii et al. [23].

**Database search with experimental database.** An additional database search was applied with a newly implemented *G. mellonella* database obtained from transcriptome analysis [13]. The transcriptomic data were translated into the single letter amino acid code, manually implemented in a FASTA file and used the same way as standard database entries for the SEQUEST database searches with BioWorks. The database included 96756 entries, mainly short sequences (64% of all entries had a chain length of less than 50 amino acids).

***De novo*** **sequencing.** Manual *de novo* sequencing was performed with accurately measured masses. Additionally, the composition-based *de novo* sequencing approach was applied using the computer program *Peptide Composer 1.0* [20], [24]. Calculations of amino acid compositions of peptides were performed using accurately measured mass spectra with a tolerance of 2 ppm for FTICR and 4 ppm for FTOT mass spectra, both for precursor ions and fragment ions. (The isobaric peptides leucine and isoleucine could not be differentiated by exact mass measurements due to their identical elemental composition.)

### Synthesized peptides

After sequence determination, peptides were custom-synthesized by *GL Biochem (Shanghai Ltd.,* China*)* with a purity of over 85%.

### Bioactivity tests

Last instar *G. mellonella* larvae, weighing between 250–350 mg, were used in this study for determining the immune stimulation. TenµL of collected fractions or 20 µL of synthesized peptide solutions (20 mM), respectively, were injected dorsolaterally into the hemocoel of the larva using 1 mL disposable syringes and 0.4×20 mm needles mounted on a microapplicator. After injection, the larvae were incubated at 32°C in darkness. 24 hours post injection, hemolymph was collected to investigate the immune stimulation.

As a simple read-out system to determine the immune-stimulatory activity of samples, we used the inhibition zone assay using freeze-dried *Micrococcus luteus* [7], [25]. The inhibition zone assay against living bacteria such as *Micrococcus luteus* is a standard assay which is widely used in microbiology to qualitatively and quantitatively determine the activity of soluble antimicrobial compounds. The latter are pipetted into wholes which have been punched in the agar inoculated with bacteria. The sample defund into the agar around thereby inhibiting the growth of the bacteria. The diameter of the clear zones without growing bacterial colonies can be measured and subsequently quantified by using a calibration curve with a standard antibiotic such as gentamycin. Accordingly, the anti-bacteria activity in samples can by quantified in gentamycin equivalents. Briefly, seven milliliter of *M. luteus* agar consisting of 1% high-purity agar-agar (*Carl Roth*, Karlsruhe, Germany), 5 mg/mL freeze-dried *M. luteus* (*Sigma*, Taufkirchen, Germany), and 67 mM potassium phosphate buffer (PBS) pH 6.4 were placed onto petri dishes (Ø100 mm). Holes with a diameter of 4 mm were filled with 3 µL hemolymph and incubated for 24h at 37°C. Lysozyme activity was quantitatively determined (units/mL) by establishing a calibration curve from standard chicken lysozyme (*Sigma*, Taufkirchen, Germany).

For validating the potency of identified peptidic danger signals to elicit immune responses we used living *M. luteus* as an indicator organism in the inhibition zone assay because bacterial growth inhibition indicates the presence of induced antibacterial peptides, whereas the lytic zone assay using freeze-dried *M. luteus* indicates enzymatic digestion by lysozyme. Synthetic peptides were dissolved in sterile saline solution (120 mM NaCl) with an end concentration of 20 mM and 20 µL were injected into larvae. For each peptide 6 individual larvae were used. The anti-*M. luteus*-activity was calculated to gentamicin equivalents (inµg/mL) using a calibration curve with gentamicin as previously described [10], [26]. Saline solution (peptide solvent) was used as control, and injected in the larvae following the same procedure as for the tested peptides. Average value and standard deviation for 6 individual animals were calculated for each peptide. Statistically significant differences between activities of larvae injected with peptides and control were determined using Students *t*-test.

## Results and Discussion

**Figure 1:**
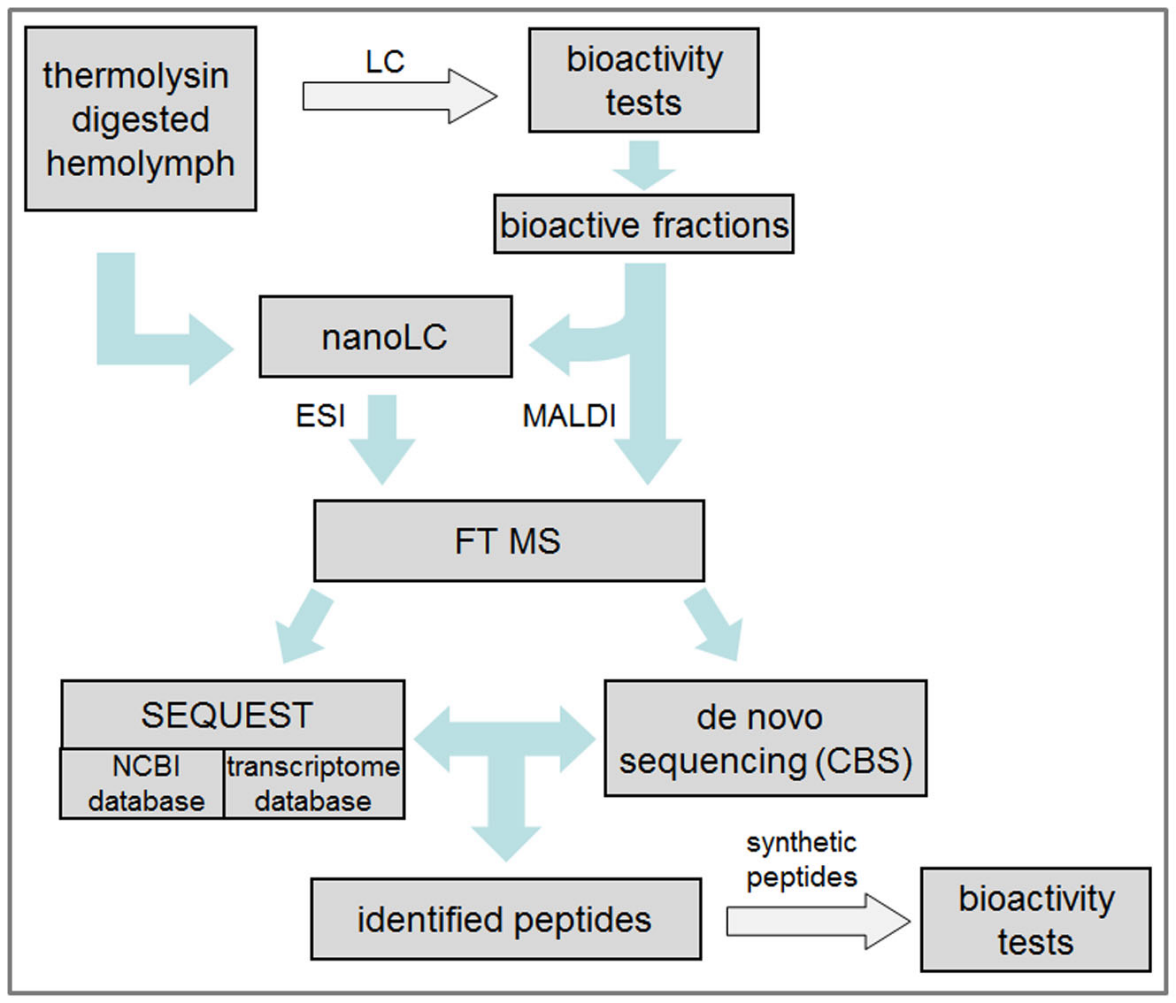
Workflow for the identification of bioactive peptides. On the one hand the thermolysin-digested hemolymph sample was analyzed directly by nanoHPLC-FTMS. On the other hand the thermolysin-digested sample was pre-fractionated using a HPLC-system and the collected samples were tested for bioactivity for a more detailed analysis of immuno-relevant peptides.

Figure 1 shows the workflow for the combined approach used for the identification of bioactive peptides. The thermolysin-digested hemolymph sample was measured directly by nanoLC-FTMS. Second, the hemolymph sample was fractionated and resulting fractions showing lysozyme inducing activity were analyzed in more detail by nanoLC-FTMS and MALDI MS. Data analysis was performed by SEQUEST database search and *de novo* sequencing. Identified peptides were tested individually for immune stimulation by synthetic analogues.

### Fractionation of hemolymph sample and initial bioactivity tests

Initial HPLC-MS measurements revealed that the thermolysin-digested sample (< 3 kDa) was too complex for the direct determination of bioactive compounds. For this reason the bulk sample was fractionated by HPLC and collected fractions were tested with a *lytic zone assay* to screen for elevated bioactivity. Details on chromatographic fractionation and activity tests can be found in the supporting information (File S1). Two fractions, termed A and B, in which the highest immune-stimulatory activity was detected were subjected to a second round of HPLC fractionation in order to further decrease the complexity. The resulting sub-fractions of these separation steps were tested again for immune-stimulatory activity. This procedure resulted in 4 sub-fractions for fraction A and 2 sub-fractions for fraction B. The tests of these sub-fractions showed that sub-fraction A1 and B1 had the highest activity and were therefore subjected to more detailed analysis.

### Peptide identification by mass spectrometry

The hemolymph samples were measured by nanoHPLC coupled to FTMS instruments. The acquired data was analyzed using standard database searches as well as experimental databases derived from transcriptome data. Bioactive fractions were analyzed in detail by nanoHPLC-FTMS and additionally by MALDI Orbitrap measurements. Peptide sequence analysis was finally extended to database-independent *de novo* sequencing methods. Identification results are reported for the bulk sample and for bioactive fractions separately, in the following. A list of all identified peptides can be found in the supporting information (Table S1).

**Database search with standard database.** One of the standard procedures in proteomics for characterization of peptides is high performance liquid chromatography coupled to mass spectrometry followed by protein database searches. In this work HPLC-FTMS measurements of the thermolysin-digested hemolymph sample were performed with database search using SEQUEST. For this purpose all rudimentary available protein sequence entries from the public database NCBI (162 protein entries) of *G. mellonella* were used.

Database search of the bulk sample with the NCBI database led to the identification of 75 peptides. The majority of these identified peptides were obtained by HPLC-MS/MS measurements where the precursor ion was measured in the FT cell with high resolution, and the five most intense ions of this survey scan were fragmented and detected in the ion trap.

Most of the identified peptides could be assigned to abundant hemolymph proteins such as hexamerin, apolipophorin and transferrin precursor. The number of identified peptides was relatively low, especially when compared with database searches of fully-sequenced species where in one run several hundred peptides can be identified. This was due to the limited number of protein entries in the database for *G. mellonella* and showed the necessity of further identification methods. The determination of a *false discovery rate* (FDR) that is common in database search studies was only partly possible, because of the small size of the used databases. An FDR of 2,7% was determined by searching a decoy database that was generated by reversing the sequences of the original NCBI database [27].

SEQUEST database search was also performed with nanoHPLC-MS measurements of the bioactive fractions A, B and the sub-fractions A1 and B1 (which had the highest bioactivity in the initial activity tests). Five peptides could be identified in this manner in the fraction B. These peptides were VV-9, IE-8, FN-9, IN-10 and LY-11 (see Table 1 for sequence information). Of these peptides IE-8 and LY-11 were also identified in the hemolymph bulk measurements discussed above. The NCBI database searches of fraction A and sub-fractions A1 and B1 showed no peptide identification.

**Table 1:** Peptides identified in the bioactive fractions.

Fraction	Sequence	[M+H]^+^_exp._	Abbr.	Used method	Ionization/
					Fragmentation
B	**IYHKPTTE**	988.50713	IE-8	NCBI DB	ESI/CID
B	**IKANAPQAEN**	1055.54729	IN-10	NCBI DB	ESI/CID
B	**FQATNDNKN**	1051.47905	FN-9	NCBI DB	ESI/CID
B	**LKTKNPSPDTY**	1263.65666	LY-11	NCBI DB	ESI/CID
B	**VDGKSAPNV**	886.46198	VV-9	NCBI & transcriptome DB	ESI/CID
B1	**APPSGPAAPPAKTP**	1258.67646	AP-14	transcriptome DB	ESI/CID
B1	**SRPSPNYP**	917.44540	SP-8	*de novo*	ESI/HCD
A1	**ERRG**	517.28360	EG-4	*de novo*	MALDI/HCD
A1	**KAERK**	631.38722	KK-5	*de novo*	MALDI/HCD

Listed are: the amino acid sequence of the peptides, the fraction in which the peptide was identified, and the method that was used for identification.

CID: Collision induced dissociation.

HCD: Higher-energy collisional induced dissociation.

DB: Database.

**Database search with experimental database.** A recently established comprehensive transcriptome of *G. mellonella* [13] was used as a database in order to identify additional peptides. Database search of the bulk sample with this experimental database resulted in 38 identified peptides. The decoy database searches of three HPLC-MS runs using the reversed database resulted in a FDR rate of 29%. These high values are most likely due to the fact that most of the database entries in the experimental database were short and therefore tended to lead to more hits with the decoy database.

The HPLC-MS measurements of the bioactive fractions were also analyzed by database search using the experimental database. In fraction B1 only one peptide could be identified by this approach: APPSGPAAPPAKTP (AP-14). In fraction B peptide VV-9 could be identified that was also identified with the standard database approach. No peptides were identified in fraction A and sub-fraction A1. The total number of peptides identified with the experimental database was 40.

***De novo*** **sequencing.** *De novo* sequencing of peptides allows identifying peptides without prior knowledge about possible amino acid sequences. Therefore this approach was used in order to identify peptides which are not included in the NCBI or transcriptome database. Data analysis by *de novo* sequencing was always performed using high resolution and high mass accuracy measurements, both for precursor and fragment ions. In doing so, it was possible to determine amino acid-specific increment masses within the MS/MS spectra. For example lysine and glutamine have the same nominal mass, but can easily be differentiated by accurate mass measurements due to their mass difference of 36 mDa.

Twenty-two peptides were identified using the *de novo* sequencing approach in the bulk sample. Nine of this set were identified by manual *de novo* sequencing of non-significant SEQUEST database search hits (Peptide Probability above threshold of 0.001).

**Figure 2:**
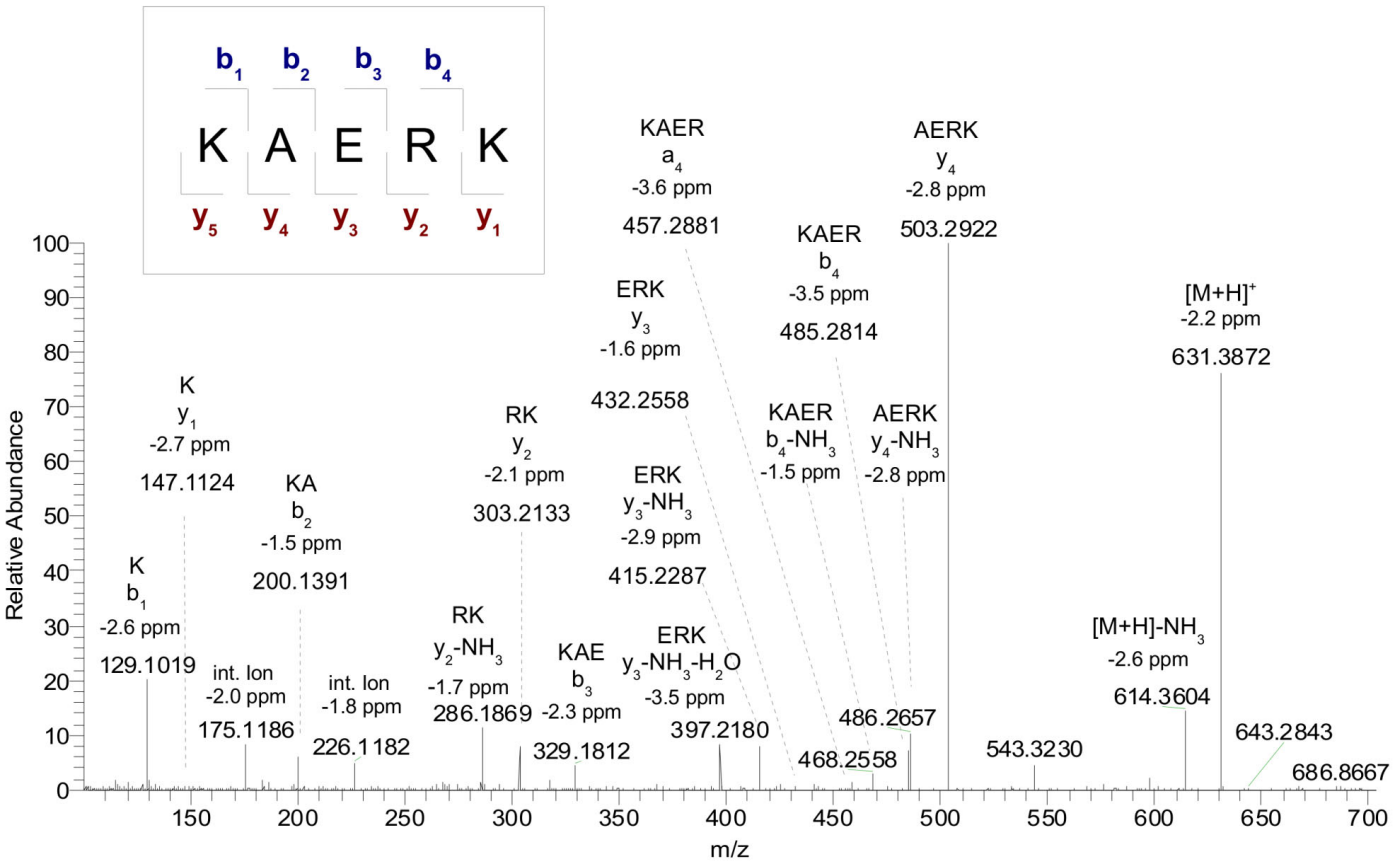
MALDI-Orbitrap MS/MS measurement with higher-energy collisional dissociation mode (HCD). The [M+H]^+^ precursor ion at m/z 631.3872 was detected in the bioactive fraction A1. The presented peptide sequence KAERK was determined by *de novo* sequencing (CBS).

The *de novo* sequencing approach led to no identification of peptides in the bioactive fractions. Therefore additional experiments were performed that implemented matrix-assisted laser desorption/ionization (MALDI) and the higher-energy collisional dissociation device (HCD) as a fragmentation technique. This fragmentation method allows the fragmentation in a dedicated quadrupolar collision cell, which results in a lower mass cut-off for fragmentation ions. An example of a peptide identified by this approach is discussed in the following. In sub-fraction A1 two peaks, m/z = 517.28360 and m/z = 631.38722, could be sequenced by the composition based sequencing approach (CBS) (see [20], [24] for more details). The MS/MS spectrum of precursor ion m/z = 631.38722 using HCD fragmentation is shown in Figure 2. For the characterization of this peptide the first step was the determination of the amino acid composition. The detected mass of m/z = 631.38722 corresponds to 11 possible amino acid compositions within an accuracy of 3 ppm. By checking against the fragment ions this value could be reduced to 4 possible amino acid compositions (A,A,S,V,K,R), (G,A,S,L,K,R), (A,A,V,T,K,R) and (A,K,K,E,R). In the MS/MS spectrum two neutral mass losses indicated the loss of glutamic acid (single letter code: E) with Δm/z = 129.0425 Da (between fragment ions m/z = 432.2558 and m/z = 303.2133) and Δm/z = 129.0421 Da (between m/z = 329.1812 and m/z = 200.1391), respectively. On the other hand, there was no indication for a loss of serine, threonine, valine or leucine. This information was used to determine the amino acid composition, as only one of the 4 remaining possibilities contains glutamic acid. The only remaining amino acid composition could therefore be determined as (A,K,K,E,R). In the second step of the composition-based *de novo* sequencing the peptide sequence was determined by scoring the agreement between observed and expected fragment ions of permuted sequence propositions. For that all fragment ion signals of the MS/MS spectrum (Figure 2) were used. The results of the *de novo* (CBS) sequencing showed that the sequence KAERK (abbreviation 'KK-5' in Table 1) was matched with the highest CBS score value. The peptide sequence of precursor ion m/z = 517.28360 was identified using the same approach as ERRG (EG-4). Three peptides were identified by *de novo* sequencing in the bioactive sub-fractions. Peptide SP-8 was identified in sub-fraction B1 in addition to the two peptides EG-4 and KK-5 discussed above. In total 25 peptides were identified by *de novo* sequencing.

**Figure 3:**
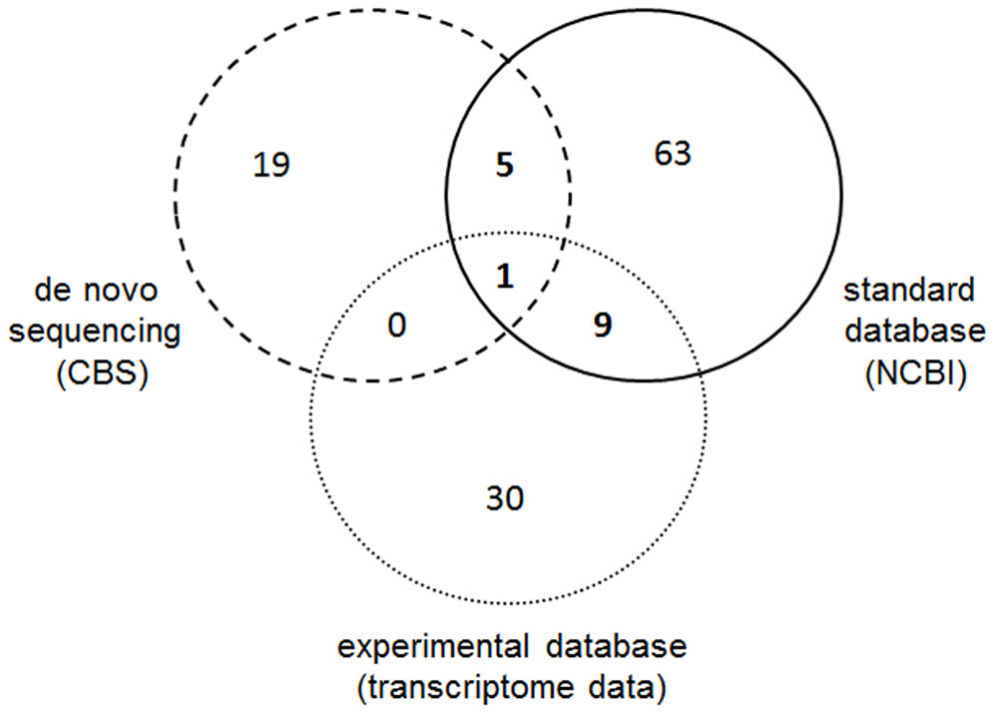
Venn diagram comparing identified peptides obtained by using different approaches. In total, 127 peptides were identified. 25 peptides were identified with the *de novo* approach, 78 with the standard database search and 40 with the experimental database.

**Comparison of identification methods.** The different approaches for the identification of peptides were summarized in a Venn diagram shown in Figure 3. Nineteen peptides were exclusively identified using the *de novo* sequencing (CBS) approach, 63 peptides could only be identified using the standard NCBI protein sequence databases of *G. mellonella*. The database search with the experimental database resulted in the identification of 30 additional peptides. Nine peptides were found in the database searches both with the NCBI and experimental databases. Five peptides were identified with *de novo* and NCBI database search. Consequently most peptides were identified by only one method. Only one peptide (IKIPAPYE) was identified with all three approaches. These results show that each sequencing approach has specific advantages and limitations. The combination of the used methods led to the identification of 127 peptides in total. Most of these peptides could be identified using the NCBI database search. In contrast, the number of peptides identified with the *de novo* approach in the bulk sample was lower. It has to be mentioned, however, that identification with the *de novo* (CBS) sequencing approach is not finally completed. Due to the fact that highly resolved MS spectra were analyzed manually, there is still the possibility of identifying additional peptides. Furthermore, reliability of the *de novo* sequencing results was higher compared to the database search results, because fragment ions were all assigned based on accurate mass measurements. The FDR rate of the search with the experimental database was high, indicating that there were also false positives among these.

Of the 127 peptides that were identified in total, 9 peptides were identified in the fractions with increased bioactivity. These peptides are summarized in Table 1. Within this set, 4 peptides were identified in fraction B using the standard NCBI database search, whereas one peptide (VV-9) was identified both with the standard and experimental database. Two peptides each were identified in sub-fraction A1 and B1. Three peptides (EG-4, KK-5 and SP-8) were identified by the *de novo* approach, whereas one peptide (AP-14) was determined by database search with the experimental database. Using the standard database search alone would have led to no results in the sub-fractions. Inclusion of MALDI and HCD was also necessary in order to identify the peptides in the sub-fractions. These results show that different ionization techniques and different approaches for peptide analysis had to be used for the identification of a broad set of peptides. All of the identified peptides in the bioactive fractions were of particular interest and had to be validated and tested for bioactivity as discussed in the following section.

**Figure 4:**
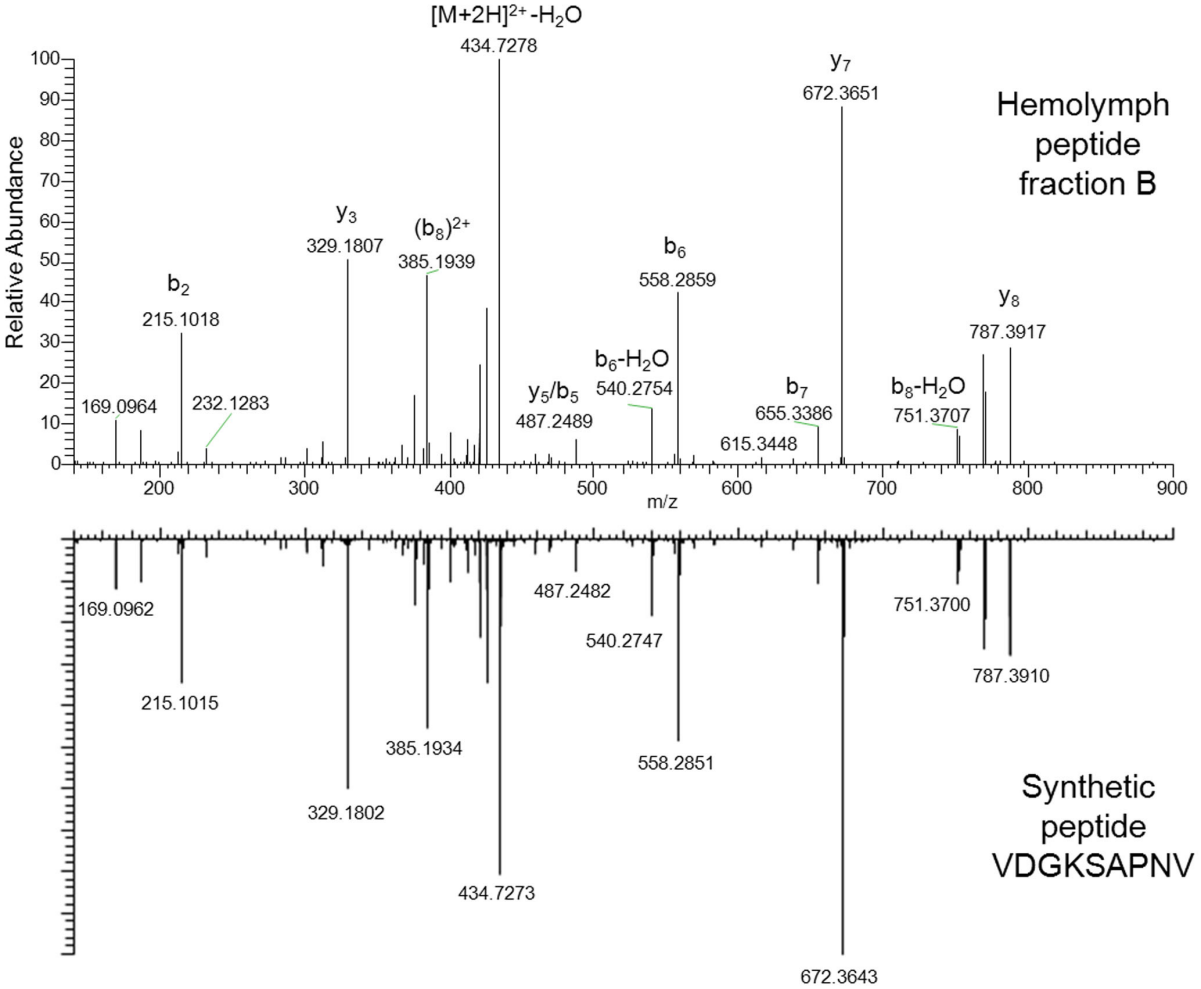
Validation of the identified peptide VV-9 in hemolymph fraction B. The MS/MS spectrum of the precursor ion m/z 886.46 was acquired with accurate mass using CID(35) as fragmentation technique. The sequence of this peptide was determined as VDGKSAPNV by database search. The fragmentation pattern of the synthetic peptide is in good accordance with the MS/MS spectrum of the identified peptide in the bioactive fraction.

**Confirmation by synthetic peptides.** To validate the sequence of the peptides identified in the bioactive fractions summarized in Table 1, synthetic analogs were synthesized. Figure 4 shows the comparison of the FT-MS/MS spectra of peptide VV-9 detected in the hemolymph fraction and its synthetic analogue. Fragmentation experiments for validation were performed the same way as for the hemolymph sample. The fragment ions of the identified peptide and of the synthetic analogue, matched all with high accuracy thus confirming the initial identification.

Seven peptides could be validated by accurate mass measurements for precursor ion and fragment ions. Two peptides identified by the database search, FN-9 and AP-14, were confirmed by accurate precursor ion mass and fragment ions measured in the ion trap. MS/MS spectra of these peptides can be found in the supporting information (File S2). Consequently all peptide sequences identified in bioactive fractions shown in Table 1 were confirmed by MS/MS measurements of synthesized peptides.

### Bioactivity tests of identified peptides

The peptides identified by our MS approaches have been postulated to represent putative danger signals resulting from digestion of hemolymph by microbial metalloproteinases. In order to validate their immune-stimulatory activity we produced synthetic analogues which were injected into last instar larvae of *G. mellonella* to determine their capacity to induce immune response in vivo. As a simple read-out system we used the inhibition zone assay against living *M. luteus* to determine antibacterial activity in the hemolymph [25].

**Figure 5:**
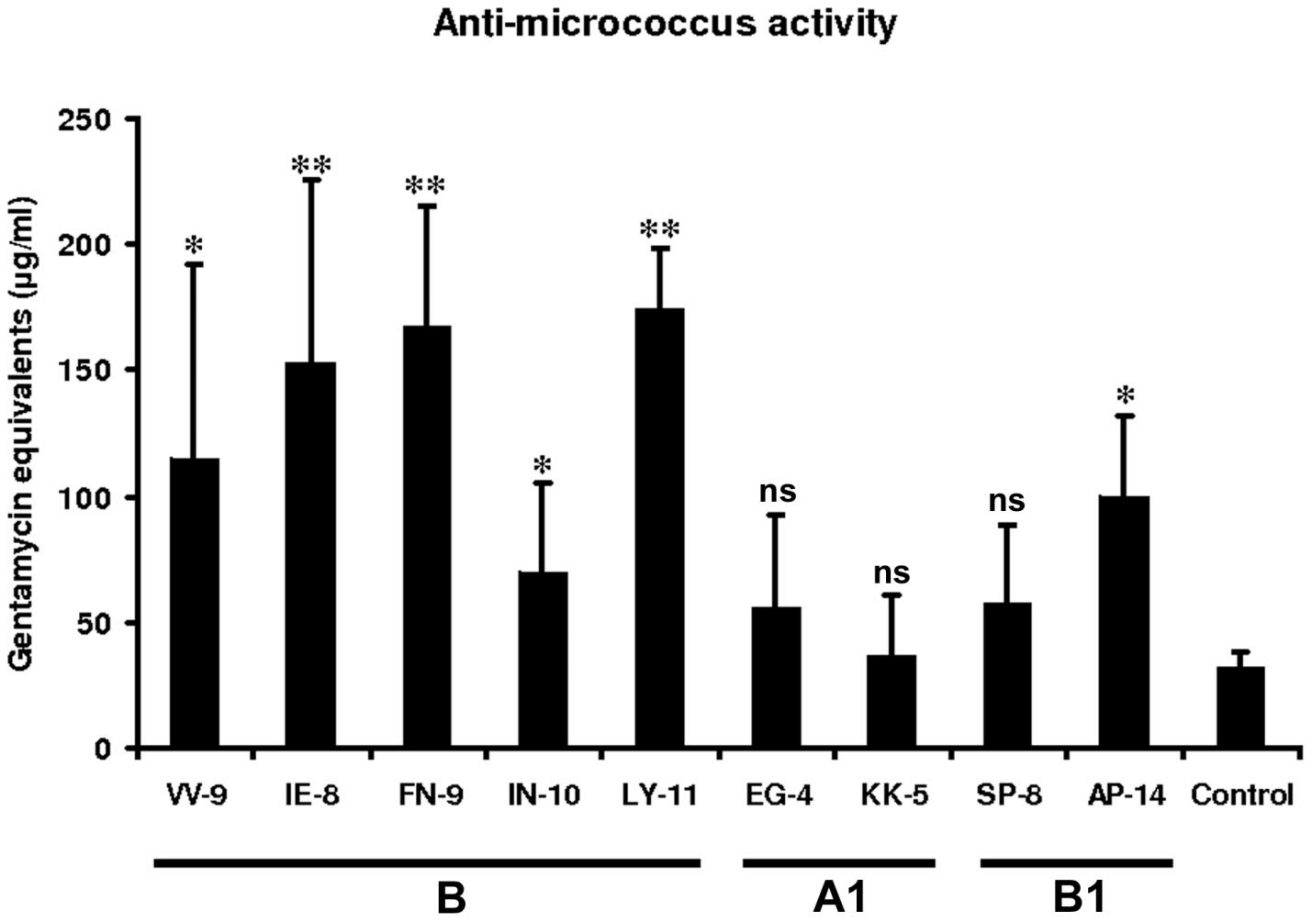
Bioactivity test of the identified peptides against (living) Micrococcus bacteria. The activity is shown in Gentamycin equivalents inµg/mL. Peptides VV-9, IE-8, FN-9, IN-10, LY-11 and AP-14 exhibit significantly higher immune-stimulatory activity than the used solvent control. Peptide KK-5 shows no activity compared to the solvent control, in contrast. EG-4 and SP-8 show slightly elevated immune activity. Statistically significant differences between activities of larvae injected with peptides and control were determined using Students *t*-test and are indicated by * (p<0.05) and ** (p<0.005), (n = 4-6 for each peptide).

Interestingly, not all synthetic peptides turned out to exhibit immune-stimulatory activity when injected (Figure 5). In comparison with control injections with saline alone, the peptides VV-9, IE-8, FN-9, IN-10, LY-11 and AP-14 induced significantly higher anti-*M. luteus*-activity. On the other hand peptides KK-5, EG-4 and SP-8 did not induce a significant response. Therefore, we conclude that only particular peptidic hemolymph protein fragments can function as danger signals when released upon metalloproteinase-mediated hydrolysis.

The larvae of the lepidopteran *G. mellonella* have been established as a powerful model host for pathogens of insects or humans and as a source for novel anti-infective therapeutics [28]. In addition, they were used to demonstrate for the first time that the presence of microbial metalloproteinases alone is sufficient to induce potent innate immune responses [7]. Thermolysin is the prototype metalloproteinase belonging to the M4 family which encompasses many virulence factors of human pathogens [9]. Thermolysin-mediated hydrolysis of hemolymph proteins from *G. mellonella* results in formation of peptidic fragments which, when injected, can elicit immune responses that are qualitatively and quantitatively comparable with those observed upon injection of bacterial cell wall components such as LPS [10].

Peptide analysis and identification in our study was more complicated in comparison to standard proteomics experiments due to several factors. Firstly, lacking a sequenced genome of *G. mellonella* it was difficult to characterize these putative danger signals by standard proteomics approaches. However, fully sequenced insect systems such as *Drosophila* and *Tribolium* are very small and therefore it is difficult to obtain hemolymph samples in sufficient quantities (for mass spectrometric analysis and bioactivity tests). Furthermore we have discovered the first specific peptidic inhibitor of metalloproteinases in *G. mellonella*. This so-called IMPI is currently being developed as new second generation antibiotic to cure symptoms caused by thermolysin-like metalloproteinases during infections in humans [28]. Secondly, peptides produced by thermolysin are more difficult to ionize than those resulting from other enzymes. Trypsin, as a digestion enzyme which produces ions which are much easier to ionize, would not be representative for the activity of microbial pathogens. It was shown previously that trypsin does not induce immune response in *Galleria mellonella* [7]. Finally, thermolysin digestion was performed at moderate enzyme concentration and for 1 hour only, which results in incomplete digestion of hemolymph proteins. A longer incubation time and/or higher thermolysin concentrations would result in a higher number of peptides. But this situation would not be comparable with in vivo conditions during infection of *G. mellonella* where only limited amounts of thermolysin-like metalloproteinases are produced, plausibly to avoid activation of host immune responses [29]. In addition it is known from our previous studies that stronger or total digestion of hemolymph proteins does not necessarily increases their immune-stimulatory activity [7].

To account for these specific challenges, we applied in this study high resolution mass spectrometry combined with *de novo* sequencing of peptides generated by metalloproteinase-mediated digestion of hemolymph proteins from *G. mellonella*. This approach led to the identification of several peptides in bioactive fractions. Following a manual inspection of LC-MS/MS data, we estimate that the two sub-fractions A1 and B1 contain about 10 peptides each (see supporting information File S3 for details). The full sequence of 2 peptides each was identified in these fractions and partial sequences were obtained for other peptides. In order to validate the postulated function of the *de novo* sequenced peptides as danger signals we produced synthetic analogues which were injected into *G. mellonella* larvae to assess their capacity to induce innate immune responses. The latter are characterized by the release of antimicrobial peptides among which some exhibit activity against living *Micrococcus luteus* [*28*]. Strikingly but in accordance with our expectation, it turned out that not all peptidic fragments exhibit immune-stimulatory activity. Only the peptides designated as VV-9, IE-8, FN-9, IN-10, LY-11 and AP-14 were confirmed as elicitors of innate immune responses when injected.

We therefore postulate that the release of metalloproteinases produced by invading pathogens and parasites results in generation of these danger signals which, in turn, induce innate immune responses [29]. The formation of these peptidic danger signals can only be detected by direct (mass spectrometric) analysis on the peptide level. Transcriptomic approaches would only detect the endogenous precursor peptides/proteins of these digestion products. In this context it is important to note that *G. mellonella* is the only known animal which is capable to produce a specific peptidic inhibitor against microbial metalloproteinases [30]. This insect metalloproteinase inhibitor (IMPI) was originally discovered in and purified from hemolymph of immune-stimulated larvae [31], and mediates reportedly feedback-loop regulation of harmful microbial metalloproteinases [32].

Consequently, we have identified peptides which are generated in *G. mellonella* under presence of microbial metalloproteinases, and which mediate the activation of immune responses encompassing the synthesis and release into the hemolymph of both antimicrobial peptides and the IMPI. Regarding the high *in vivo* toxicity of thermolysin-like metalloproteinases, the characterized peptidic products of their activity can be considered as danger signals because they can set the immune system into alarm. This efficient mechanism providing the ability to sense invading pathogens or parasites by the activity of their virulence-associated enzymes [29] adds to the sophisticated strategies determining the tremendous evolutionary plasticity of insect immunity [33].

## Conclusions

In this study we used high-resolution mass spectrometry to identify peptidic hemolymph protein fragments which are generated in the hemolymph of the model host *G. mellonella* when harmful microbial metalloproteinases belonging to the M4 family, with thermolysin as the prototype, are present. Addressing the lack of a genome sequence we complemented the rudimentary NCBI database with a transcriptome database and *de novo* sequencing approaches for peptide identification. This approach led to the identification of 9 potentially bioactive peptides. These (tentative) results were validated by studying synthesized peptide analogues. Detailed MS/MS experiments confirmed the amino acid sequence of all 9 peptides. Six out of 9 peptides identified in the bioactive fractions exhibited immune-stimulatory activity when injected in *Galleria* larvae. These six peptidic elicitors of immune responses are postulated to function as danger signals indicating the presence of microbial metalloproteinases and mediating their regulation by specific inducible metalloproteinase inhibitors. Our results suggest that the validity of the immunity-related danger model proposed by Matzinger can be expanded beyond mammals to include insects. The detailed molecular pathways and importance of this process will have to be investigated in follow-up studies.

## Supporting Information

Table S1 List of identified peptides.
(DOC)

File S1 **Chromatographic fractionation and activity tests of hemolymph.**
(DOC)

File S2 **MS/MS spectra of peptides identified in hemolymph and of the corresponding synthetic peptide standards.**
(DOC)

File S3 **Estimating number of peptides in bioactive fractions.**
(DOC)
