# Supplementary material for: High-Resolution Mass Spectrometry Driven Discovery of Peptidic Danger Signals in Insect Immunity
Source: PLoS One. 2013 Nov 26;8(11):e80406. doi: 10.1371/journal.pone.0080406 (PMC3841204; doi:10.1371/journal.pone.0080406)
Supplement: Table S1 — List of identified peptides. (DOC) [file pone.0080406.s001.doc]

**Table S1: List of identified peptides**

| **Sample** | **m/z (1+)** | **Sequence** | **Peptide Probability** | **Method** | **ppm** |
| --- | --- | --- | --- | --- | --- |
| bulk | 433.24441 | LGFP |  | de novo | 0.27 |
| bulk | 463.25539 | LAYP |  | de novo | -0.65 |
| bulk | 473.29688 | LLDL |  | de novo | 0.16 |
| bulk | 491.25052 | LDFP |  | de novo | -1.05 |
| bulk | 502.28794 | LSVPS |  | de novo | -1.63 |
| bulk | 514.32418 | (L/V)VPS |  | de novo | -1.31 |
| fraction A1 | 517.28360 | ERRG |  | de novo | 0.97 |
| bulk | 520.31355 | LIQF |  | de novo | -1.17 |
| bulk | 530.29682 | FPGLP |  | de novo | 0.89 |
| bulk | 591.35088 | LFVNV |  | de novo | -1.40 |
| bulk | 594.31313 | (T/Y)LPT |  | de novo | 0.34 |
| bulk | 601.32011 | LGVPES | 6.78E-04 | de novo/DB | -1.60 |
| bulk | 607.29196 | VGGMPF | 6.49E-05 | de novo/DB | -1.87 |
| bulk | 612.30366 | VFPSY |  | de novo | -1.46 |
| bulk | 621.32488 | LDLFN |  | de novo | -1.04 |
| fraction A1 | 631.38722 | KAERK |  | de novo | 2.13 |
| bulk | 651.35488 | MLYPK |  | de novo | -2.24 |
| bulk | 657.34627 | LGELPE |  | de novo | -1.38 |
| bulk | 662.31444 | LAASWD | 1.57E-04 | de novo/DB | -0.07 |
| bulk | 663.33489 | E.IFGEPT.L | 3.58E-04 | DB | -0.11 |
| bulk | 676.40308 | N.VGYKIP.L | 4.73E-04 | DB/exp. DB | -0.35 |
| bulk | 689.34947 | V.LPAPYE.I | 6.74E-04 | DB | 1.45 |
| bulk | 689.36981 | N.LLVTDE.I | 4.36E-04 | DB | 2.59 |
| bulk | 689.39820 | LGKFPAG |  | de novo | -0.19 |
| bulk | 693.34388 | LFPWM | 8.96E-04 | de novo/DB | -1.43 |
| bulk | 705.37982 | N.IESIPF.I | 2.42E-04 | DB | 2.76 |
| bulk | 717.38055 | R.IPPIYD.V | 3.82E-04 | DB | 1.69 |
| bulk | 729.37921 | S.ISVEGPAG.A | 3.50E-04 | DB | -2.01 |
| bulk | 738.36499 | Q.LSYTPTG.V | 3.55E-05 | DB | 2.51 |
| bulk | 749.34810 | G.ASTTPSW.F | 9.82E-05 | DB | -2.21 |
| bulk | 771.36223 | Y.ITNTGEH.V | 6.59E-05 | DB | 1.20 |
| bulk | 778.35698 | A.VNDDSKT.V | 2.38E-04 | exp. DB | 0.97 |
| bulk | 785.48881 | S.IDVLGRL.I | 7.32E-04 | DB | -1.07 |
| bulk | 797.37881 | V.INNPDPQ.F | 1.33E-04 | exp. DB | 0.49 |
| bulk | 811.42087 | N.VLSGSTTF.I | 7.36E-04 | DB | -1.56 |
| bulk | 813.43674 | S.VGLGEIPE.L | 4.44E-04 | DB | -1.81 |
| bulk | 819.42273 | P.FDEIPAK.L | 3.74E-05 | de novo/DB | 2.37 |
| bulk | 821.49986 | T.VTLPHRV.L | 8.14E-04 | DB | -0.81 |
| bulk | 827.45211 | G.LGEIPELG.L | 2.30E-04 | DB | -1.45 |
| bulk | 831.48374 | D.FRLPNGK.I | 3.84E-04 | DB | -0.23 |
| bulk | 847.41210 | L.LDDRTDL.F | 8.77E-04 | DB | 4.12 |
| bulk | 847.49489 | K.LIDIFIN.M | 4.39E-04 | DB | -2.96 |
| bulk | 850.41415 | I.LEDKSGTT.V | 7.17E-04 | DB | 1.30 |
| bulk | 855.49230 | L.IEQLKPAG.F | 2.90E-04 | exp. DB | 1.34 |
| bulk | 877.40575 | N.AGASTTPSW.F | 2.57E-04 | DB | -0.82 |
| bulk | 882.46825 | I.LRGDIPDP.L | 8.41E-04 | DB | -0.33 |
| fraction B | 886.46198 | E.VDGKSAPNV.I | 1.09E-09 | DB/exp. DB | 1.01 |
| bulk | 889.39410 | D.FVPADPED.M | 2.01E-06 | DB/exp. DB | -0.35 |
| bulk | 896.44722 | F.VINNPDPQ.F | 7.79E-04 | exp. DB | 1.93 |
| bulk | 899.46427 | P.LSNPPQFP.I | 1.10E-04 | DB | -2.36 |
| bulk | 900.49467 | T.LGFPVDRP.L | 9.83E-06 | DB | -0.99 |
| bulk | 901.50401 | G.IVLPAPYE.I | 7.65E-04 | DB/exp. DB | -1.18 |
| bulk | 909.57747 | D.IGVVPIPKS.L | 1.45E-04 | exp. DB | -0.76 |
| bulk | 910.42099 | L.FFHPHEP.I | 8.00E-04 | DB | -0.41 |
| bulk | 911.52045 | G.LTDLPQKP.A | 2.02E-04 | exp. DB | -0.87 |
| bulk | 913.46075 | F.VGLPENPTS.V | 1.68E-04 | exp. DB | 1.95 |
| bulk | 915.50433 | YQVPRGVP |  | de novo | 0.36 |
| fraction B1 | 917.44540 | SRPSPNYP |  | de novo | 2.33 |
| bulk | 930.52692 | G.IKIPAPYE.I |  | de novo/  DB/exp DB | 2.74 |
| bulk | 934.48818 | P.VVETFPDK.F | 8.74E-06 | DB | -0.17 |
| bulk | 936.56265 | G.ITVTLPHR.V | 3.40E-06 | DB | -0.13 |
| bulk | 938.44857 | Q.LVLDYSDN.I | 6.15E-05 | exp. DB | -2.15 |
| bulk | 939.51554 | H.VADNIPIPT.L | 8.77E-05 | DB/exp DB | -1.03 |
| bulk | 949.47430 | P.LNVNTQNF.L | 2.94E-06 | DB | -0.56 |
| bulk | 957.41650 | R.IDEPGNSPE.I | 4.85E-04 | DB | -0.54 |
| bulk | 960.46143 | T.ILTEGNEQG.V | 1.88E-05 | DB | 1.92 |
| bulk | 960.50057 | K.LLDDRTDL.F | 3.84E-04 | DB | -0.96 |
| bulk | 966.50542 | G.VTSRFPYP.I | 3.00E-05 | DB | -1.11 |
| bulk | 968.48439 | T.ALRDPAYY.M | 5.04E-04 | DB | -0.81 |
| bulk | 977.62658 | N.ILKTPRHL.L | 2.64E-04 | DB | -1.14 |
| bulk | 983.55202 | G.LQEKLPQQ.F | 8.08E-04 | exp. DB | 3.07 |
| bulk | 986.56695 | M.IRP*PLFEV.F | 7.19E-04 | exp. DB | 3.47 |
| bulk | 986.56855 | D.IRIPPIYD.V | 4.62E-05 | DB | -1.63 |
| fraction B/bulk | 988.50694 | Q.IYHKPTTE.V | 8.62E-07 | DB | 2.91 |
| bulk | 995.55377 | Q.ILRGDIPDP.L | 1.38E-06 | DB | -1.76 |
| bulk | 1018.43054 | A.IGAPSSNEDE.L | 5.61E-06 | exp. DB | 1.78 |
| bulk | 1019.62588 | A.VIVVHKDLP.I | 2.70E-06 | DB/exp DB | -1.07 |
| bulk | 1035.63141 | G.ITVTLPHRV.L | 2.02E-08 | DB | -0.46 |
| bulk | 1042.55327 | T.VTLPDGSTRP.L | 5.19E-05 | DB | -0.50 |
| bulk | 1045.45586 | Y.ISEYHAPND.V | 2.33E-06 | DB | 2.54 |
| fraction B/bulk | 1051.47905 | E.FQATNDNKN.V | 5.18E-05 | DB | 1.20 |
| fraction B/bulk | 1055.54656 | D.IKANAPQAEN.L | 1.82E-08 | DB | 1.37 |
| bulk | 1060.53172 | K.FVGLPENPTS.V | 6.74E-04 | exp. DB | -0.73 |
| bulk | 1071.56511 | S.LKKPDDIEN.L | 9.38E-04 | exp. DB | 2.76 |
| bulk | 1073.54274 | L.LYHNGIPYP.V | 2.96E-06 | DB | -1.20 |
| bulk | 1076.58442 | S.LILEDKSGTT.V | 1.90E-07 | DB | -0.96 |
| bulk | 1084.53823 | V.ILHRSDTQD.I | 4.76E-04 | DB | -0.06 |
| bulk | 1085.63680 | D.IRIPPIYDV.F | 2.29E-07 | DB | -1.33 |
| bulk | 1090.66223 | Q.AVIVVHKDLP.I | 7.10E-04 | DB | -0.30 |
| bulk | 1106.51190 | N.VVDENPDYR.V | 8.82E-04 | exp. DB | -0.56 |
| bulk | 1111.60024 | G.LTDLPQKPAE.F | 2.55E-05 | exp. DB | -0.79 |
| bulk | 1124.65618 | L.LLPKGRVGGMP.F | 3.27E-05 | DB | 4.17 |
| bulk | 1125.61998 | A.INMPNGRKVP.L | 1.09E-04 | DB | -0.22 |
| bulk | 1136.61165 | L.IGVTSRFPYP.I | 6.26E-08 | DB | -1.56 |
| bulk | 1138.52909 | L.IRGSNASS#GLP.M | 5.14E-04 | exp. DB | -3.39 |
| bulk | 1140.55785 | I.IAKNVNQGC@H.L | 3.65E-04 | exp. DB | 2.93 |
| bulk | 1144.59042 | S.LDPFDEIPAK.L | 1.73E-04 | DB | -1.70 |
| bulk | 1145.54045 | S.VSVETQGSPQN.V | 1.62E-05 | DB | 2.49 |
| bulk | 1149.51384 | A.FRLPDDSGEGG.L | 4.92E-06 | exp. DB | 2.83 |
| bulk | 1174.60625 | N.LANEANSKSAGL.F | 1.04E-05 | exp. DB | 0.00 |
| bulk | 1186.62903 | G.LLYHNGIPYP.V | 8.39E-07 | DB | -2.96 |
| bulk | 1187.59952 | F.VSKDDHKGKSS.I | 3.63E-04 | exp. DB | 1.66 |
| bulk | 1206.67267 | E.ISEKKLPQYT.L | 3.54E-04 | DB | 0.16 |
| bulk | 1237.74692 | H.LLLPKGRVGGMP.F | 3.14E-04 | DB | -1.61 |
| bulk | 1242.52941 | E.FRTYDEPDAE.F | 1.15E-07 | DB/exp DB | -1.68 |
| fraction B1 | 1258.67646 | T.APPSGPAAPPAKTP.V | 1.31E-08 | DB | 2.03 |
| bulk | 1260.53386 | K.VVDNSDPNGQDT.V | 1.75E-04 | exp. DB | 1.55 |
| fraction B/bulk | 1263.65666 | S.LKTKNPSPDTY.V | 1.19E-06 | DB | 1.01 |
| bulk | 1271.73079 | L.LLPKGRVGGMPF.V | 1.15E-04 | DB | -1.19 |
| bulk | 1282.67586 | C.VILC@FSSLTKD.L | 8.09E-04 | exp. DB | -3.68 |
| bulk | 1282.67217 | S.VVILHRSDTQD.I | 4.56E-11 | DB | 2.20 |
| bulk | 1282.73426 | R.MSLRRYK*ILS.A | 3.96E-04 | exp. DB | -3.31 |
| bulk | 1308.75452 | Q.ILRGDIPDPLSL.I | 7.88E-06 | DB | -1.79 |
| bulk | 1333.60285 | G.VDEPRYNPTGAD.V | 9.46E-10 | DB/exp DB | -0.72 |
| bulk | 1359.60350 | K.VVDNSDPNGQDTV.I | 2.35E-04 | exp. DB | -0.90 |
| bulk | 1361.71249 | R.VEYPTNKGLPFP.I | 5.83E-05 | DB | -1.84 |
| bulk | 1384.81631 | H.LLLPKGRVGGMPF.V | 9.03E-04 | DB | -2.14 |
| bulk | 1393.73604 | M.LHNNYQVPRGVP.F | 1.59E-05 | DB | -1.82 |
| bulk | 1468.80664 | G.LQVPPVVETFPDK.F | 3.66E-06 | DB | -1.39 |
| bulk | 1501.68901 | A.VNGASHGYPVEEKD.V | 1.79E-08 | DB | 1.83 |
| bulk | 1510.67599 | A.LLCNPSHIFNSVS#.L | 4.22E-04 | exp. DB | 2.76 |
| bulk | 1532.70631 | D.LEWRT#GT#AGRLI.L | 1.68E-06 | exp. DB | -2.67 |
| bulk | 1581.84339 | C.ASLLRS#LKLMADAL.L | 4.74E-04 | exp. DB | 0.14 |
| bulk | 1581.84875 | P.LGTIPAKPSTEDPKQ.F | 8.29E-07 | DB/exp DB | -0.31 |
| bulk | 1681.68663 | K.ADTICLP*HESDM*KSC.L | 6.31E-04 | exp. DB | -3.88 |
| bulk | 1954.89379 | -.FY#MILS#RVK*AQIYF.L | 6.94E-05 | exp. DB | -1.05 |
| bulk | 2460.32102 | I.VIIFPCSSLRNINLVVDDDKSL.A | 7.06E-04 | exp. DB | -1.77 |

Leucine and isoleucine (L/I) are not differentiated in the fragmentation mass spectra.

Posttranslational modifications: * hydroxylation Δm = +15.99492, # phosphorylation Δm = +79.96633 and @ carbamidomethylation Δm = +57.02146.

All available entries for Galleria were downloaded in 2008/11/17 in the fasta-file format from the server of the “*National Center of Biotechnology Information*” NCBI (<http://www.ncbi.nlm.nih.gov/>) and used as the standard database (DB).
