# Supplementary material for: High-Resolution Mass Spectrometry Driven Discovery of Peptidic Danger Signals in Insect Immunity
Source: PLoS One. 2013 Nov 26;8(11):e80406. doi: 10.1371/journal.pone.0080406 (PMC3841204; doi:10.1371/journal.pone.0080406)
Supplement: File S1 — Chromatographic fractionation and activity tests of hemolymph. (DOC) [file pone.0080406.s002.doc]

**File S1: Chromatographic fractionation and activity tests of hemolymph**

HPLC fractionation of bulk sample:


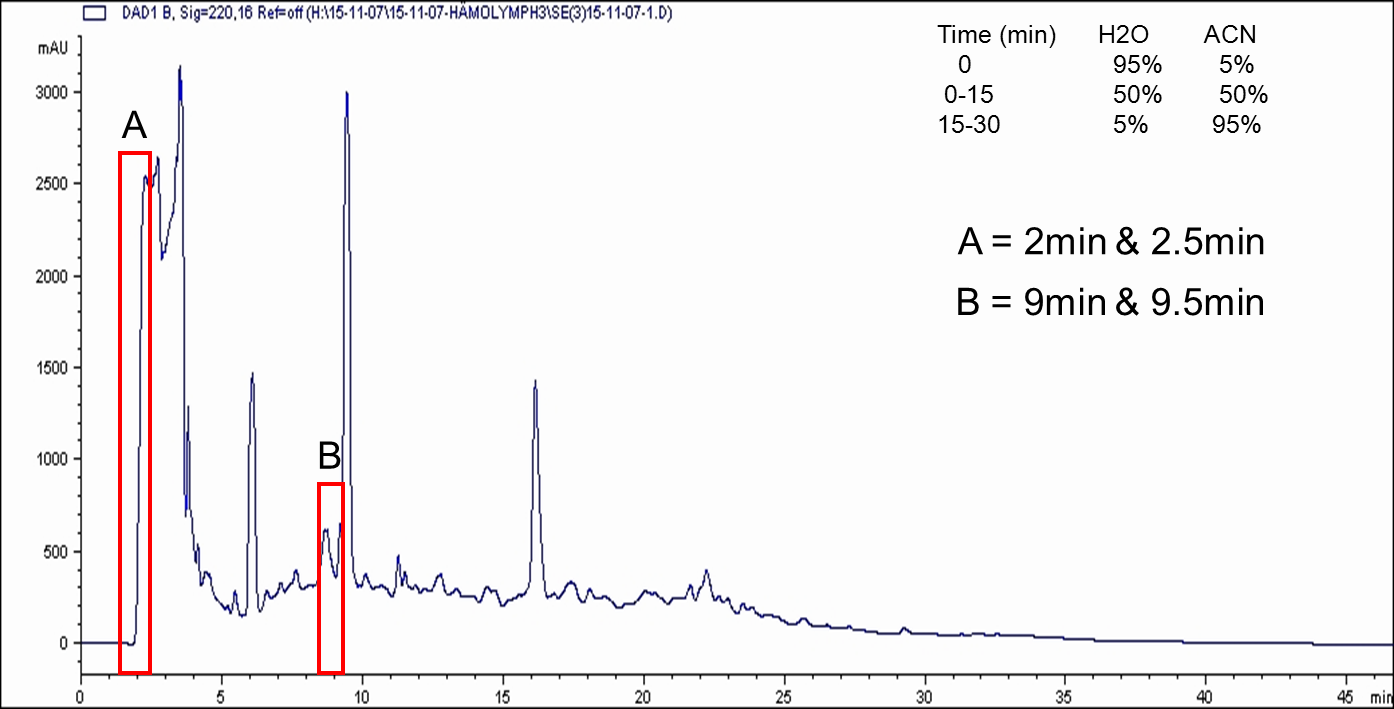


Figure (S1): HPLC-UV detection at 220 nm, injection volume 100 µL

Figure (S2): Results of the lysozyme activity tests of the separated fractions 1-21 and 31-34 in [unit/mL]. Additionally the lysozyme activity of deionized water is analyzed as control. Fractions 4, 5, 18 and 19 showed the highest bioactivity. Two fractions each were combined resulting in sample A and B, respectively, as indicated.

HPLC sub-fractionation of samples A and B


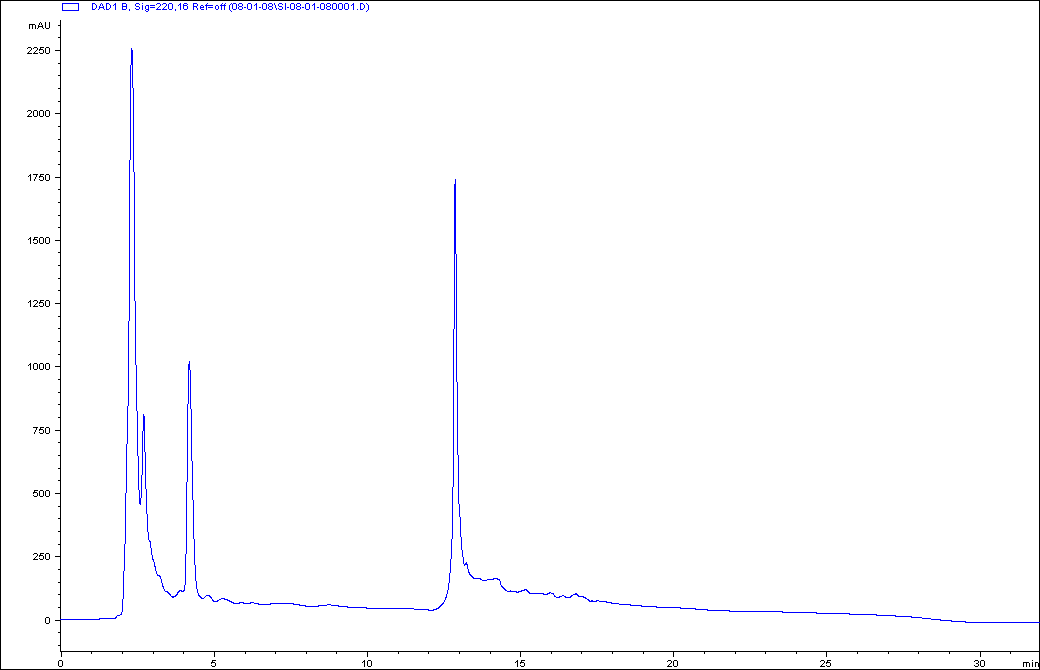


**A_2**

**A_1**

**A_3**

**A_4**

Time (min) H_2_O ACN

0 - 8 100% 0%

8-13 50% 50%

13-18 50% 50%

Figure (S3): HPLC-UV detection at 220 nm of sample A, injection volume 100 µL

Sample (A) sub-fractions

Figure (S4): Lysozyme activity obtained 24h post-injection of the sample (A) sub-fractions.


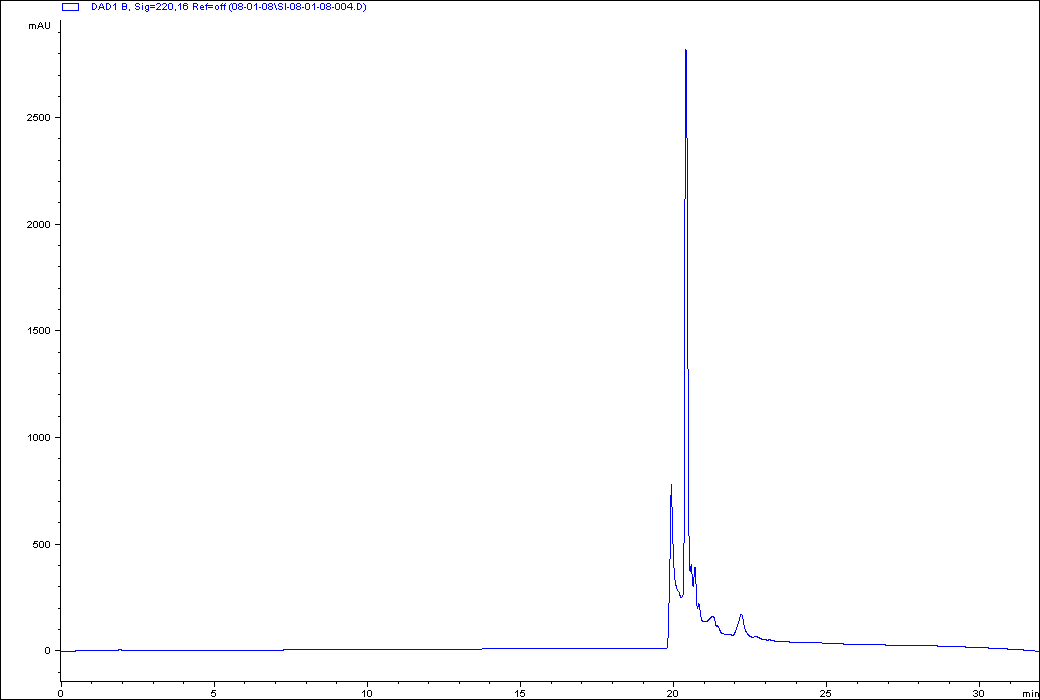


B_1

B_2

Time (min) H_2_O ACN

0 - 15 100% 0%

15-19 50% 50%

19-25 50% 50%

Figure (S5): HPLC UV detection at 220 nm of sample B, injection volume 100 µL

Sample (B) sub-fractions

Figure (S6): Lysozyme activity obtained 24h post-injection of the sample (B) sub-fractions.
