# Supplementary material for: High-Resolution Mass Spectrometry Driven Discovery of Peptidic Danger Signals in Insect Immunity
Source: PLoS One. 2013 Nov 26;8(11):e80406. doi: 10.1371/journal.pone.0080406 (PMC3841204; doi:10.1371/journal.pone.0080406)
Supplement: File S2 — MS/MS spectra of peptides identified in hemolymph and of the corresponding synthetic peptide standards. (DOC) [file pone.0080406.s003.doc]

**File S2: MS/MS spectra of peptides identified in hemolymph and of the corresponding synthetic peptide standards**


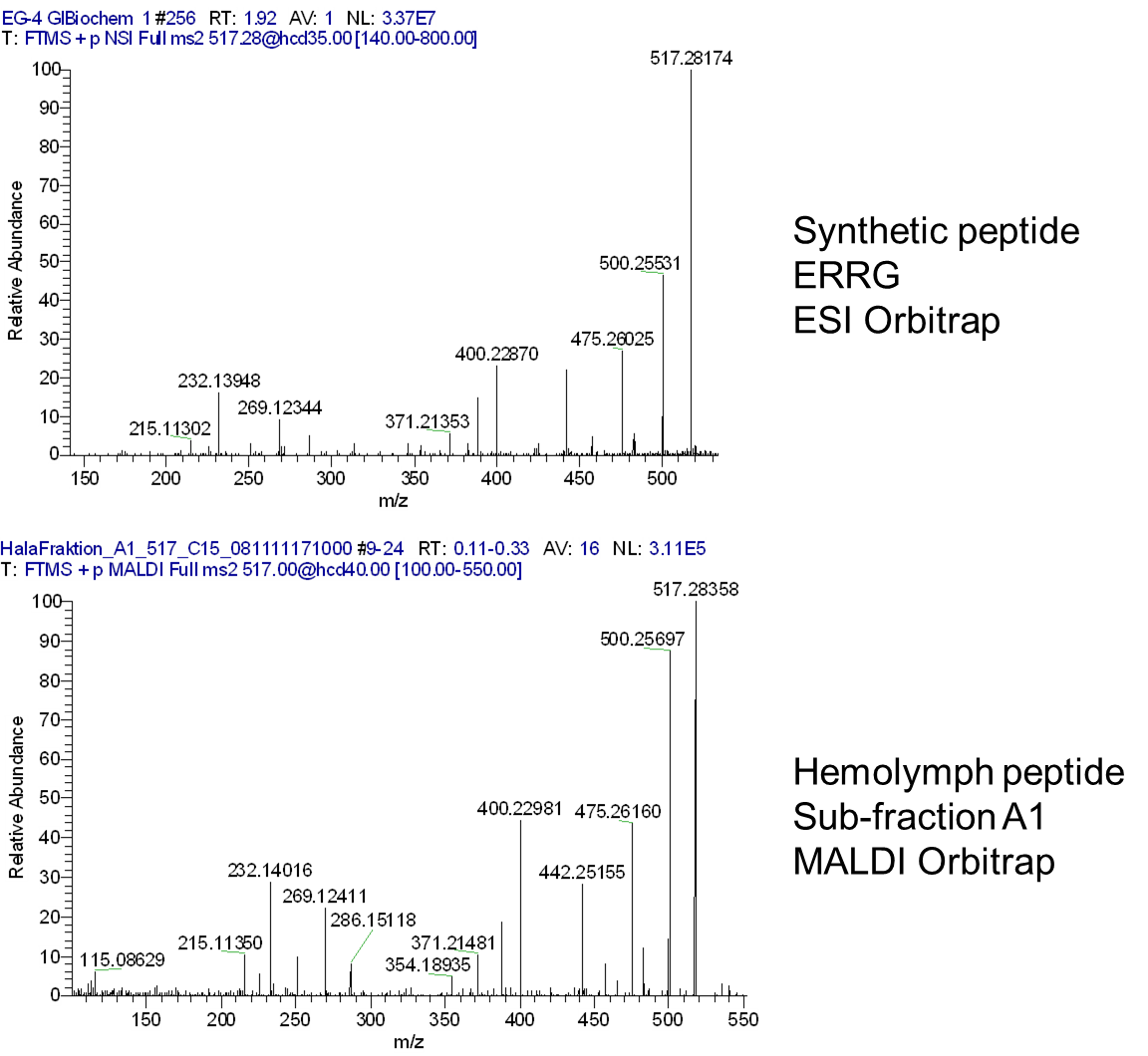


Figure (S7) MS/MS of peptide ERRG


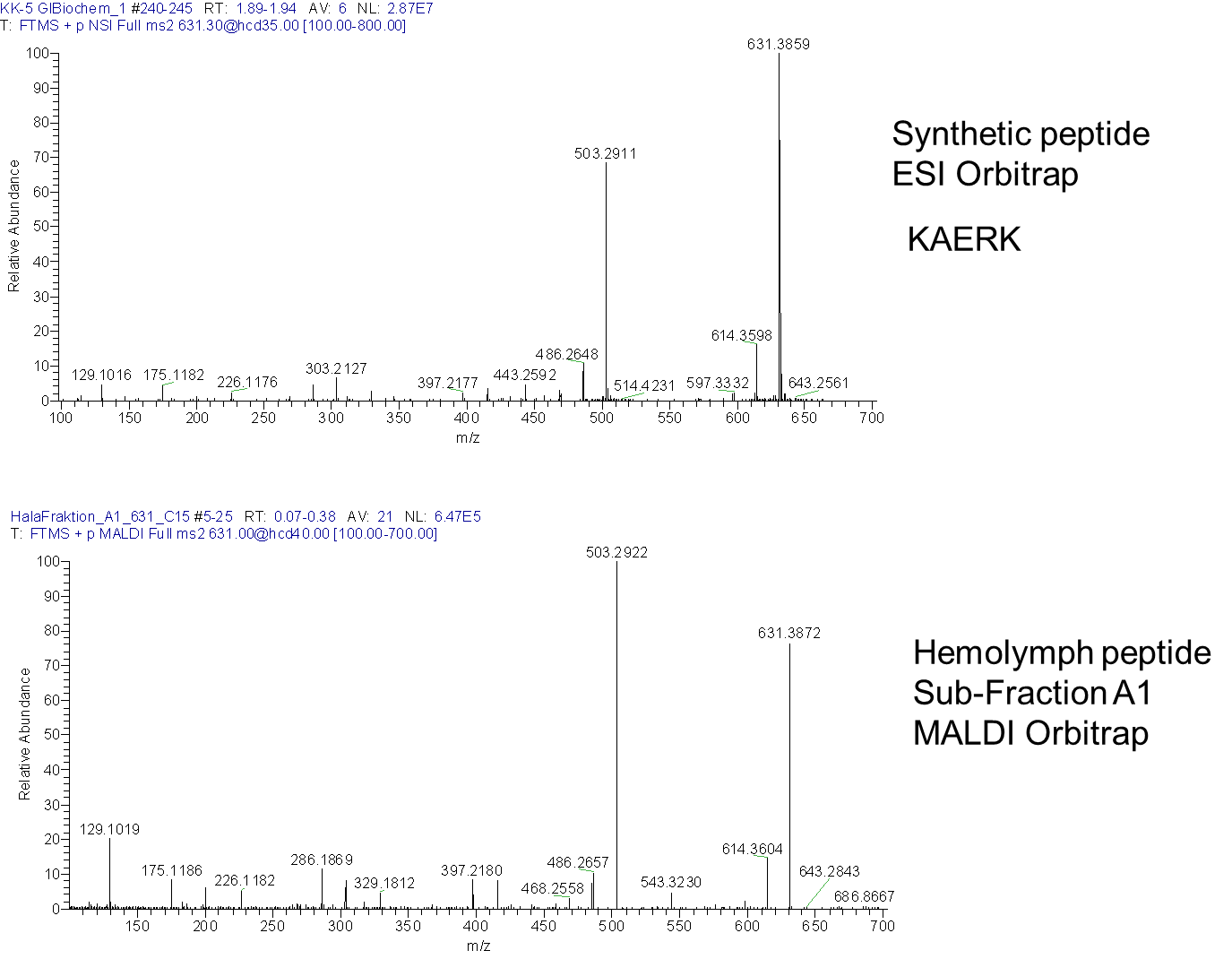


Figure (S8) MS/MS of peptide KAERK


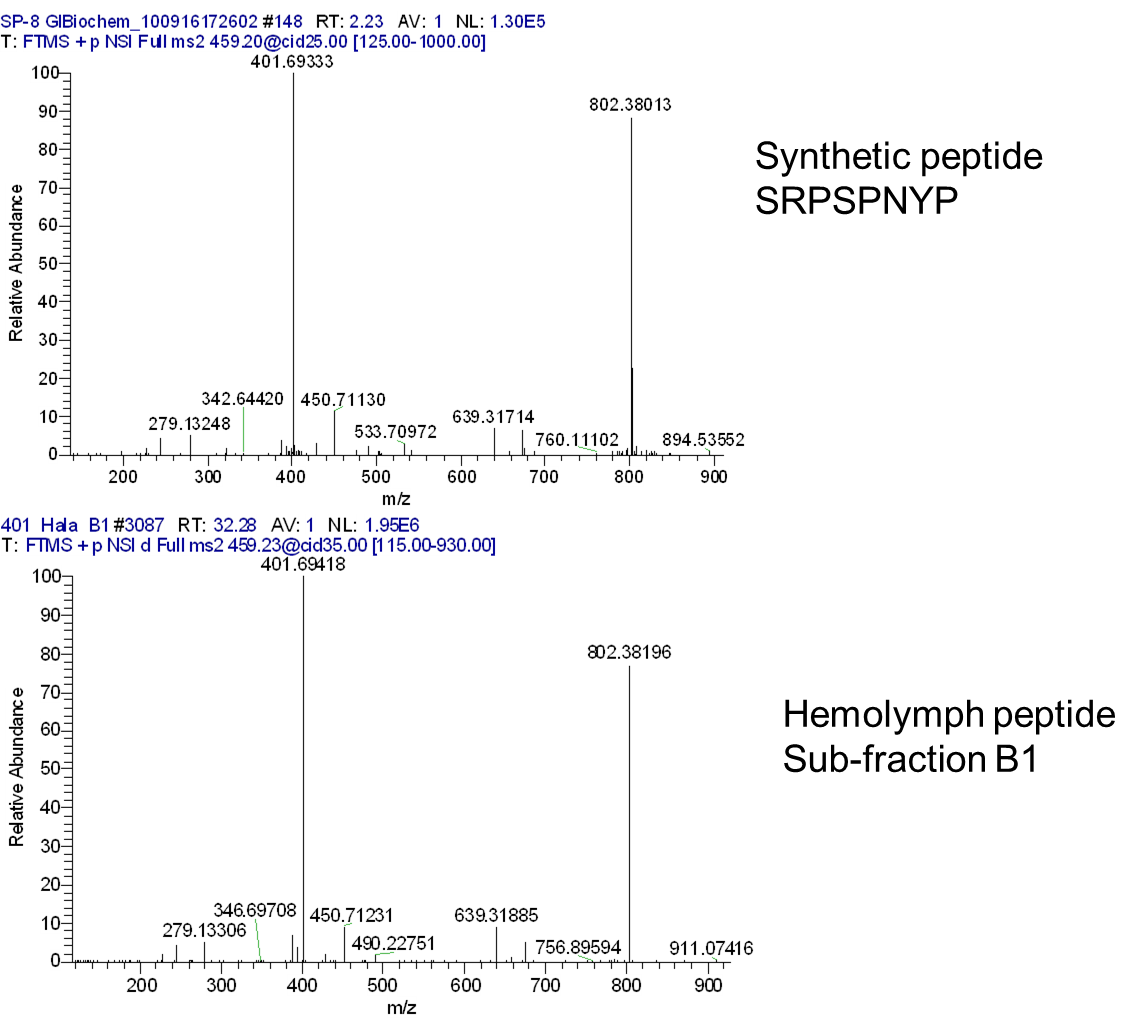


Figure (S9) MS/MS of peptide SRPSPNYP


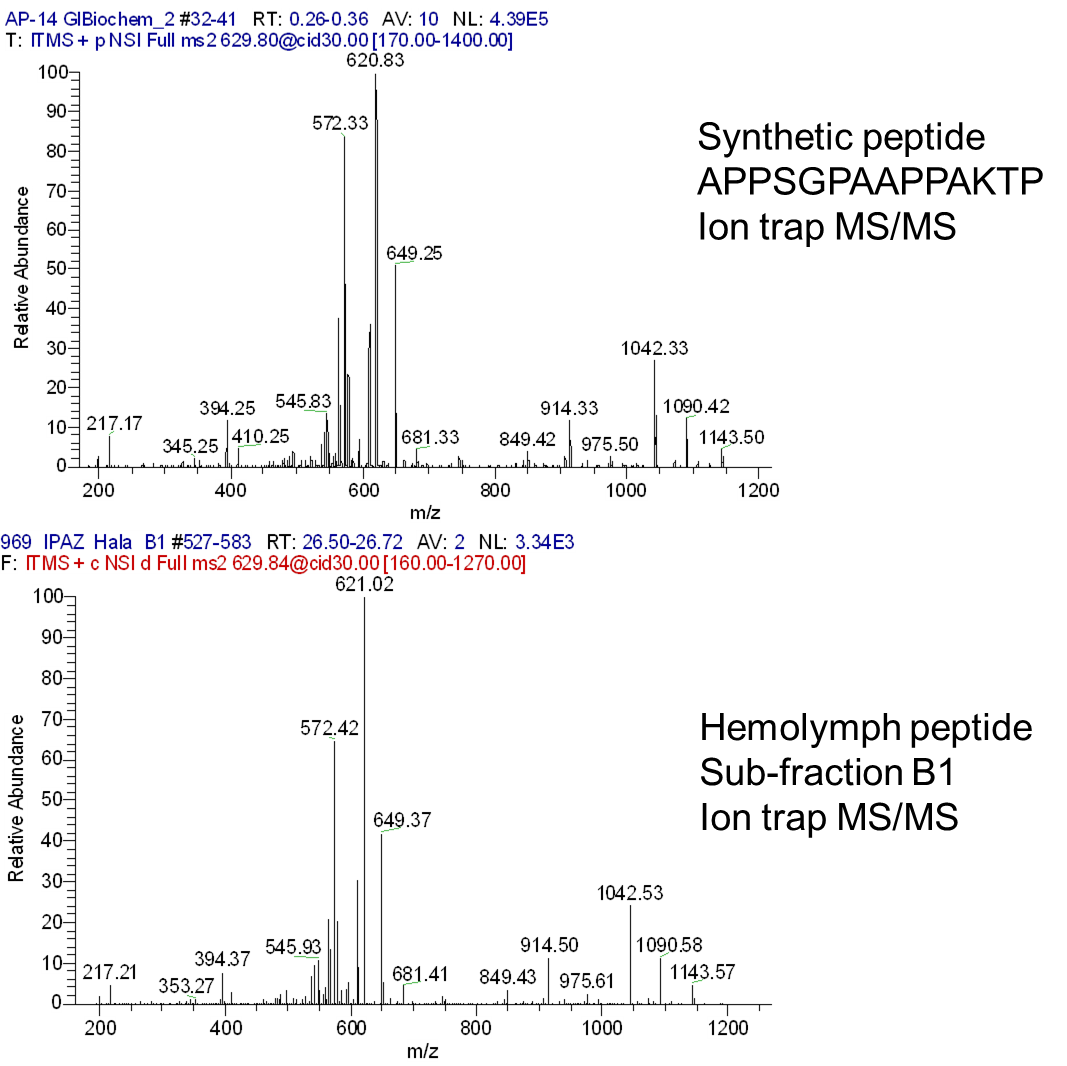


Figure (S10) MS/MS of peptide APPSGPAAPPAKTP


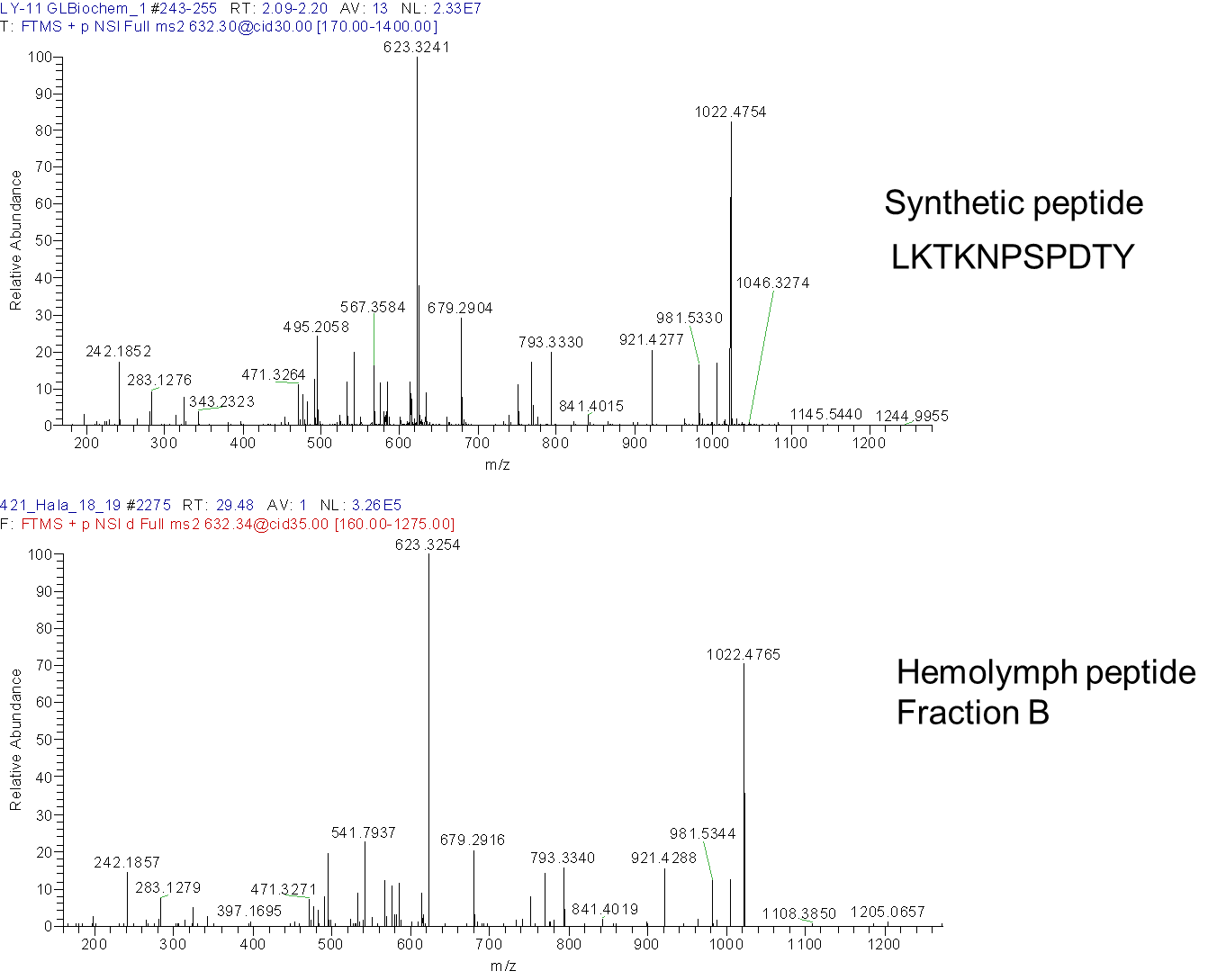


Figure (S11) MS/MS of peptide LKTKNPSPDTY


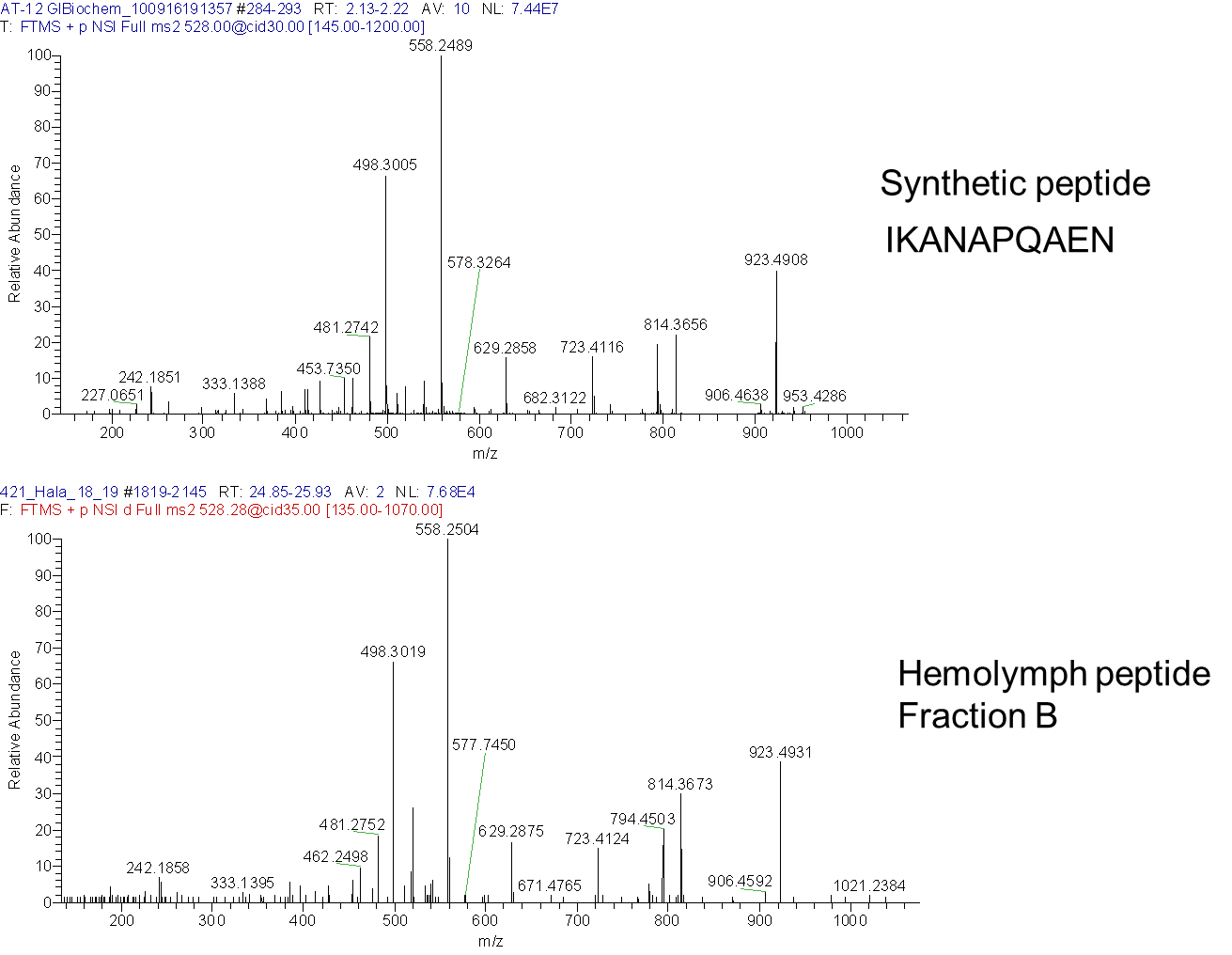


Figure (S12) MS/MS of peptide IKANAPQAEN


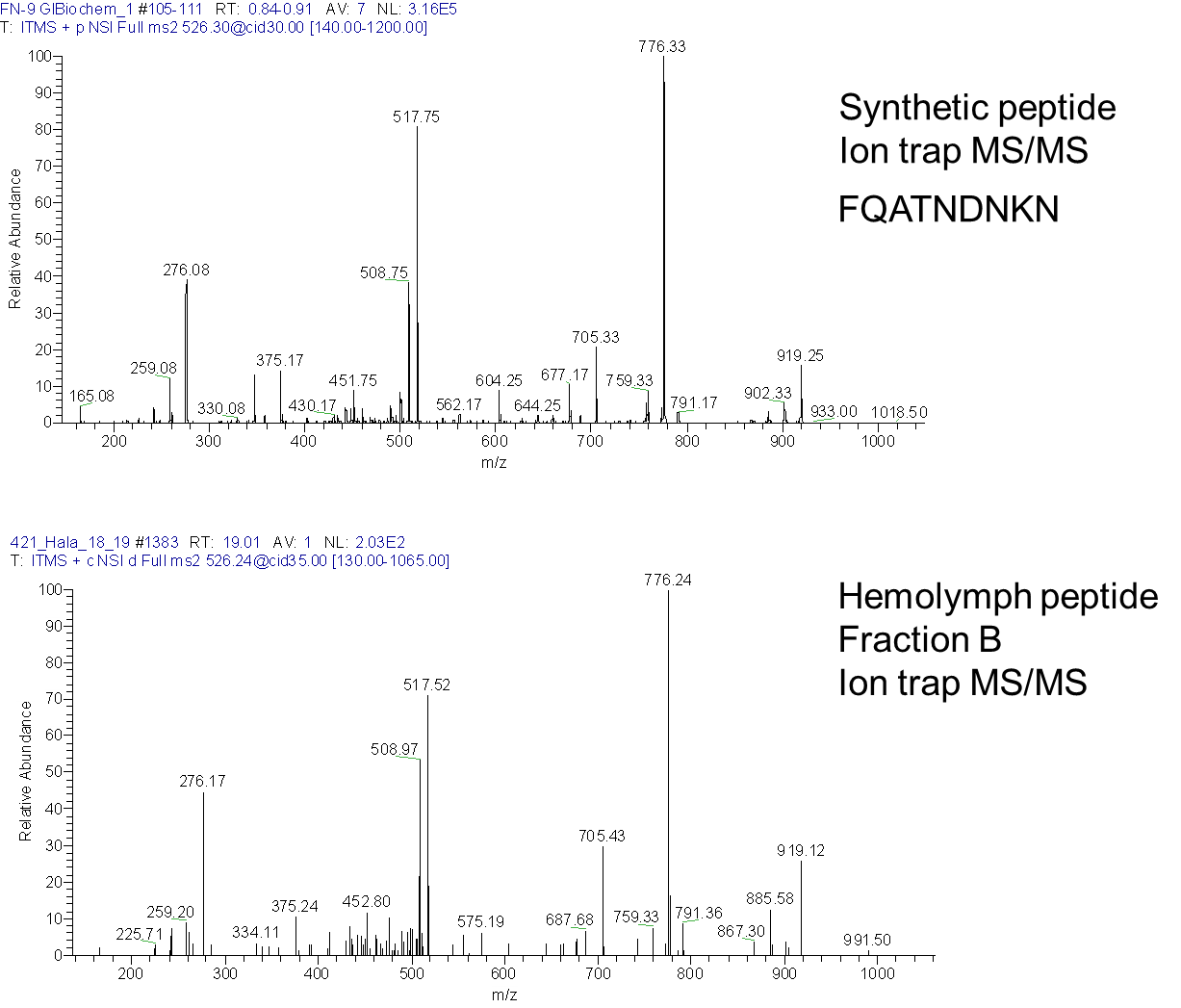


Figure (S13) MS/MS of peptide FQATNDNKN


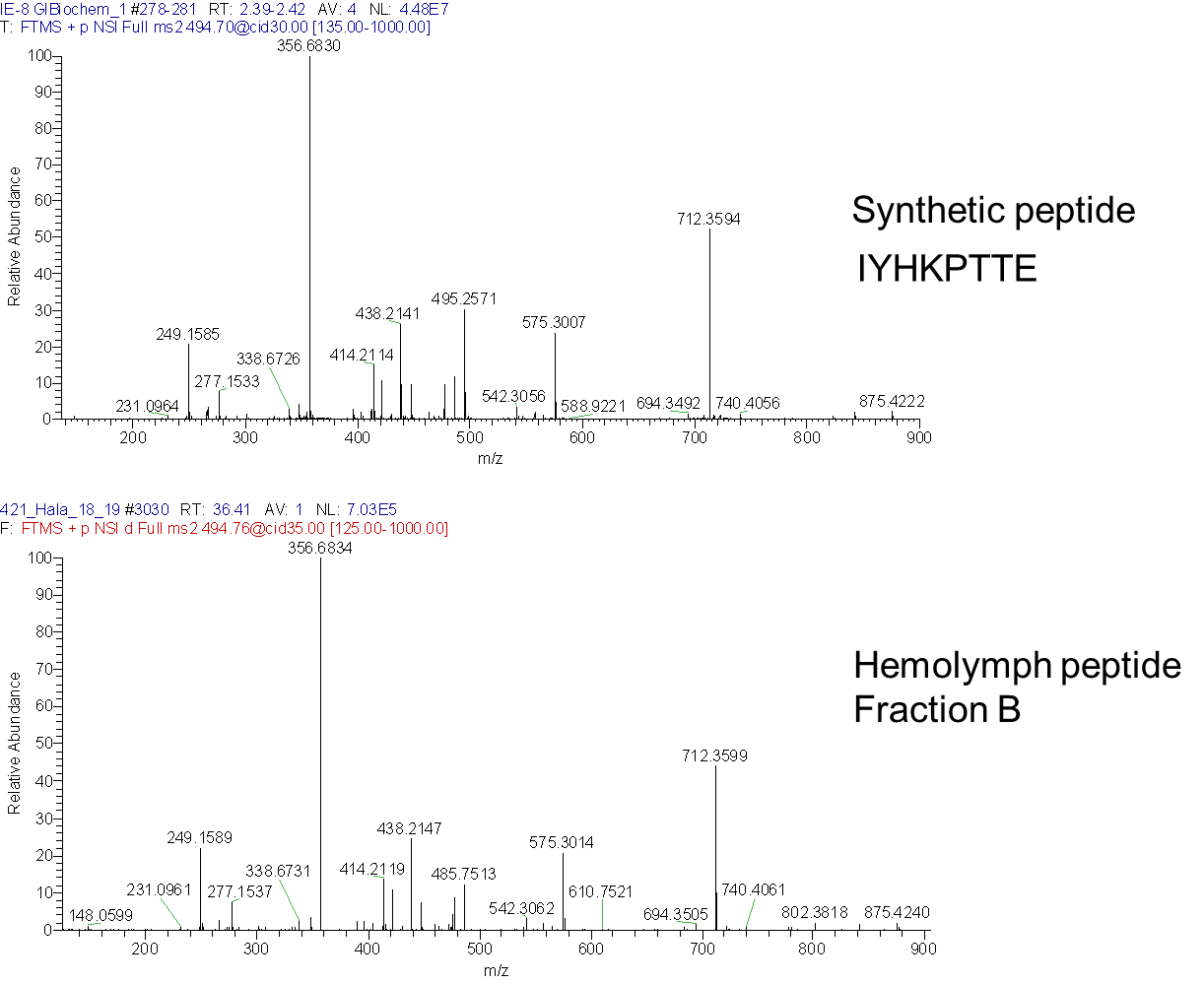


Figure (S14) MS/MS of peptide IYHKPTTE
