# Supplementary material for: High-Resolution Mass Spectrometry Driven Discovery of Peptidic Danger Signals in Insect Immunity
Source: PLoS One. 2013 Nov 26;8(11):e80406. doi: 10.1371/journal.pone.0080406 (PMC3841204; doi:10.1371/journal.pone.0080406)
Supplement: File S3 — Estimating number of peptides in bioactive fractions. (DOC) [file pone.0080406.s004.doc]

**File S3: Estimating number of peptides in bioactive fractions**

MS/MS spectra of the LC-MS/MS runs were manually inspected using the data analysis software Xcalibur (Thermo Fisher Scientific). The ‘Info Bar’ function for MS/MS spectra was used to select precursor ions. Only precursor ions in the retention time window for peptides (10 – 45 min) and with m/z > 400 were considered for this analysis. MS/MS spectra were checked for fragment ions and neutral loss reactions which correspond to amino acids. This should provide a reasonable indication of (potential) peptides. This analysis resulted in 9 and 10 potential peptides in sub-fractions A1 and B1, respectively. As mentioned above this is only a rough estimation in order to get an idea about the number of peptides.

It should also be noted again that de novo identification requires much higher quality tandem mass spectra with sufficient fragment ion signals as compared to database search. Therefore only partial or no sequence information could be obtained for some of these peptides.
